# A Systematic Review on the Effect of Transcranial Direct Current and Magnetic Stimulation on Fear Memory and Extinction

**DOI:** 10.3389/fnhum.2021.655947

**Published:** 2021-03-22

**Authors:** Vuk Marković, Carmelo M. Vicario, Fatemeh Yavari, Mohammad A. Salehinejad, Michael A. Nitsche

**Affiliations:** ^1^Department of Psychology and Neurosciences, Leibniz Research Centre for Working Environment and Human Factors, Dortmund, Germany; ^2^International Graduate School of Neuroscience, Ruhr-University-Bochum, Bochum, Germany; ^3^Department of Cognitive Science, University di Messina, Messina, Italy; ^4^Department of Neurology, University Medical Hospital Bergmannsheil, Bochum, Germany

**Keywords:** non-invasive brain stimulation, fear memory, dorsolateral prefrontal cortex, ventromedial prefrontal cortex, repetitive transcranial magnetic stimulation, fear extinction

## Abstract

Anxiety disorders are among the most prevalent mental disorders. Present treatments such as cognitive behavior therapy and pharmacological treatments show only moderate success, which emphasizes the importance for the development of new treatment protocols. Non-invasive brain stimulation methods such as repetitive transcranial magnetic stimulation (rTMS) and transcranial direct current stimulation (tDCS) have been probed as therapeutic option for anxiety disorders in recent years. Mechanistic information about their mode of action, and most efficient protocols is however limited. Here the fear extinction model can serve as a model of exposure therapies for studying therapeutic mechanisms, and development of appropriate intervention protocols. We systematically reviewed 30 research articles that investigated the impact of rTMS and tDCS on fear memory and extinction in animal models and humans, in clinical and healthy populations. The results of these studies suggest that tDCS and rTMS can be efficient methods to modulate fear memory and extinction. Furthermore, excitability-enhancing stimulation applied over the vmPFC showed the strongest potential to enhance fear extinction. We further discuss factors that determine the efficacy of rTMS and tDCS in the context of the fear extinction model and provide future directions to optimize parameters and protocols of stimulation for research and treatment.

## Introduction

Anxiety disorders are among the most prevalent mental disorders (Kessler et al., 2009; Bandelow and Michaelis, 2015). With 3.4% (264 million) of the global population affected (WHO, 2017), 16.6% lifetime prevalence (Remes et al., 2016), and an increased number of affected patients due to population aging and growth (WHO, 2017), anxiety disorders have a relevant impact on patients and societies worldwide. Accordingly, disease burden led to a total of 24.6 million years lived with this disability (YLD) in 2015 (WHO, 2017), unemployment and loss of productivity at work, reduced quality of life (Kessler et al., 2009; Simpson et al., 2010; Remes et al., 2016; Martino et al., 2019), higher risk of mortality (Van Hout et al., 2004), and vast financial burden (Andlin-Sobocki and Wittchen, 2005; Kessler et al., 2009; Wittchen et al., 2011). Analysis of the efficacy of currently available routine treatments, such as pharmacological treatment with serotonin reuptake inhibitors (SRIs), and cognitive-behavioral therapy, shows that about a fifth of the patients terminate treatment prematurely, one third is classified as non-responders, and complete recovery is uncommon (Taylor et al., 2012). The development of more efficient therapies is thus required, which could in turn lead to improved well-being of the patients, and reduction of societal costs.

A large number of studies use the fear extinction model as a model for studying the psychopathology of anxiety disorders, and therapeutic mechanisms (Milad et al., 2014; VanElzakker et al., 2014). In this model, which is based on Pavlovian fear conditioning, participants undergo different phases of learning. During the acquisition phase, a neutral stimulus (e.g., blue light) is paired with a biologically potent aversive stimulus (unconditioned stimulus (US), e.g., electrical shock) which provokes a fear response. After repeated presentation and pairing, the neutral stimulus becomes the conditioned stimulus (CS), with a potential to provoke the respective fear response on its own. In the extinction phase, participants are exposed to the CS without US, and gradually, fear responses decline and, at the end, are extinguished. Extinction recall, as the final phase, is assessed typically the following day. In this phase, participants are again exposed to the CS without US presentation in order to evaluate the retention of extinction memory. Beyond these classical protocols, recently virtual reality (VR) approaches have been introduced, which have the potential to improve the ecological validity of respective procedures (Huff et al., 2010; Maples-Keller et al., 2017).

Fear extinction is most often measured by several psychophysiological parameters, such as skin-conductance response (SCR), heart rate response (HRR), or fear-potentiated startle (FPS; electromyography), which monitor vegetative responses, i.e., enhanced sympathetic tone, to the respective stimuli. SCR, HRR, and FPS are enhanced during fear acquisition, but reduced by fear extinction (Hamm and Vaitl, 1996; Milad et al., 2005; Norrholm et al., 2006; Hein et al., 2011). Beyond these vegetative parameters, also psychological measures, such as US-expectancy ratings (i.e., the prediction that the CS would be followed by the US) are obtained, which add a cognitive component to respective outcome measures (Zuj et al., 2018). In animal models, the fear response is often measured through freezing behavior as an indicator of anxiety-like behavior (Richmond et al., 1998; Chang et al., 2009; Roelofs, 2017).

Providing an exhaustive overview of the neural circuits involved in fear acquisition and extinction is beyond the scope of this review and we will focus only on the core brain structures and their role in the above-mentioned process. More elaborated overviews can be found elsewhere (see, Herry et al., 2010; Knapska et al., 2012; Milad and Quirk, 2012; Tovote et al., 2015). Neural circuits of fear acquisition and extinction include several areas of the brain, such as the amygdala, ventromedial prefrontal cortex (vmPFC), dorsolateral prefrontal cortex (dlPFC), dorsal anterior cingulate cortex (dACC), insula and hippocampus. Different parts of the amygdala are considered as crucial for the acquisition, expression and extinction of fear (Barad et al., 2006; Myers and Davis, 2007). Signals from the US and CS converge in the basolateral complex (BLA) of the amygdala, which processes those stimuli and sends its output to the central nucleus (CEA) (Barad et al., 2006). Neurons in the central nucleus initiate physiological and behavioral response characteristics of fear (Sah and Westbrook, 2008). The intercalated (ITC) amygdala neurons are relevant for extinction of conditioned fear (i.e., ITC neurons receive information about the CS from the BLA and have inhibitory projections to the CEA) (Likhtik et al., 2008). In accordance, neuroimaging studies in humans show enhanced activation of the amygdala during acquisition of a conditioned fear response, whereas during extinction training, its activity gradually diminishes (Phelps et al., 2004; Milad et al., 2007). Therefore, it can be concluded that inputs from the US and CS converge in the BLA during fear conditioning, are processed and sent to the CEA, which initiates fear-related physiological and behavioral responses. ICT neurons of the amygdala contribute to fear extinction via their inhibitory projections to the CEA.

Apart from the amygdala, the anterior cingulate cortex (ACC) has a role in expression of fear responses. Multichannel unit recordings in animal models showed that activity of pre-limbic cortex (PL) neurons, the homolog of the dorsal ACC (dACC) in humans, correlates with the expression of fear (Burgos-Robles et al., 2009). Furthermore, the resting state metabolism of this area predicts the magnitude of conditioned fear responses (Linnman et al., 2011) in humans. In accordance, the persistence of PL responses after extinction training was associated with a failure to express extinction memory in rats (Burgos-Robles et al., 2009). Furthermore, ontogenetic methods have shown that PL activity is not critical for the expression of extinction memory (Kim et al., 2016). For the insula, higher BOLD reactivity was related to greater SCRs (Linnman et al., 2011) and greater thickness of this area was related to larger conditioned responses during fear acquisition (Hartley et al., 2011). Moreover, a meta-analysis by Stark et al. (2015) suggests that the right anterior insula could be a core region of the network undergoing changes after experiencing a traumatic or painful event.

In summary, amygdala, the ACC, and the insular cortex are crucial structures in the acquisition of aversive conditioning (see, Sehlmeyer et al., 2009). Furthermore, the hippocampus is involved in fear conditioning. It is activated during contextual and simple cue fear conditioning in humans and animals, and activates or inhibits fear expression depending on the context of learning (VanElzakker et al., 2014; Sevenster et al., 2018). Regarding the prefrontal cortex, research in animal models suggests that the dlPFC is important to promote the expression of learned fear (Morgan et al., 1993; Quirk et al., 2006). Furthermore, a recent study (Kroes et al., 2019) in six patients with dlPFC lesions and 19 control participants provided evidence that the dlPFC might be essential in providing a cognitive regulation of subjective fear to threatening stimuli. Moreover, it was suggested that the dlPFC has a role in detection of uncertainty, and similar to the insula and ACC, shows higher activity during fear conditioning, since higher activity in these areas is detected when the CS-US pairing is uncertain (Dunsmoor et al., 2007, 2008).

The vmPFC is a central area involved in mediating mechanisms of extinction learning and recall. It is activated during fear extinction, but not acquisition (Phelps et al., 2004; Milad et al., 2007). Moreover, a lesion of the infralimbic (IL) cortex, the homolog of the human vmPFC, impairs recall of fear extinction in rodents (Quirk et al., 2000), and the IL is activated during extinction recall in rats (Milad et al., 2007). In accordance, a recently conducted optogenetics study by Do-Monte et al. (2015) showed that activation, contrary to silencing, of the IL during extinction learning improves subsequent retrieval and, further, this structure is relevant for controlling the expression of fear after extinction (Kim et al., 2016). The vmPFC/IL sends direct projections to ICT neurons, and thus controls the output of the amygdala during extinction (Quirk and Gehlert, 2003; Sah and Westbrook, 2008). In accordance, Motzkin et al. (2015) found that vmPFC lesions of patients were associated with increased right amygdala reactivity to aversive stimuli, suggesting disinhibition of the amygdala. Therefore, it has been proposed that the vmPFC is responsible for top-down regulation of the amygdala (Milad et al., 2007), and that dysfunctions of vmPFC-amygdala connectivity may mediate the susceptibility to and/or maintenance of anxiety disorders (Milad et al., 2014). Moreover, the vmPFC is an important target of the hippocampus in context-dependent expression of fear extinction memory (Kalisch et al., 2006; Milad et al., 2007).

Anxiety disorders, post-traumatic stress disorder (PTSD) and obsessive-compulsive disorder (OCD), show deficits in fear extinction and functionality of the neural circuits discussed above (Milad et al., 2014). Patients with panic disorder (PD) exhibit larger SCR in response to conditioned stimuli during extinction, and maintain a more negative evaluation of CSs, as compared to healthy controls (Michael et al., 2007). In accordance, individuals with PTSD show larger responses to CSs during acquisition and extinction with respect to SCR, EMG and HRR in comparison to healthy individuals (see, Milad et al., 2014; VanElzakker et al., 2014). Impaired extinction recall is documented also in OCD patients (Milad et al., 2013; McLaughlin et al., 2015). Several studies have found alterations of specific fear- and extinction-relevant neural circuits in these diseases. For patients with generalized anxiety disorder (GAD), deficient vmPFC activity has been observed during fear-related task performance (Greenberg et al., 2013; Via et al., 2018). Individuals with PTSD show structural and functional deficits of various fear-related areas of the brain, including amygdala, PFC and hippocampus. Amygdala responsivity is positively associated with symptom severity in PTSD (Shin et al., 2006), and a smaller volume of the amygdala has been described in individuals with PTSD (Morey et al., 2012). Furthermore, during extinction recall, PTSD patients show reduced activation of the vmPFC and hippocampus, but increased activation of the dACC (Milad et al., 2009). With respect to specific phobias, patients with spider phobia show increased activity of the insula and reduced activity of the vmPFC in automatic emotion regulation (Hermann et al., 2009). In accordance, extinction-based exposure therapy of specific phobias reduces amygdala hyperactivity (Goossens et al., 2007).

Advances in neuroscience methods have the potential to enrich our understanding of the basic neural mechanisms of anxiety disorders and, consequently, lead to the development of new treatment options, and protocols. The above-mentioned studies enable the identification of candidate areas critically involved in respective processes, and thus identification of targets for new interventions such as brain stimulation approaches. In this connection, recent reviews showed that non-invasive methods of brain stimulation (NIBS), including transcranial direct current stimulation (tDCS) and repetitive transcranial magnetic stimulation (rTMS), are promising therapeutic approaches for anxiety disorders (Vicario et al., 2019, 2020a) as well as other psychiatric conditions including pediatric populations (see Vicario and Nitsche, 2013a,b, 2019; Salehinejad et al., 2019, 2020).

tDCS modulates cortical excitability with direct electrical currents that pass through the cerebral cortex (Nitsche and Paulus, 2000; Nitsche et al., 2003, 2008). Electrical currents (1 ~ 2 mA) are delivered via two or more electrodes of opposite polarities (i.e., anode and cathode) placed on the scalp. tDCS does not generate action potentials, but instead modulates resting neuronal membrane potentials at subthreshold levels (Nitsche and Paulus, 2000). Anodal stimulation increases cortical excitability, while cathodal stimulation decreases it (Stagg and Nitsche, 2011) during stimulation, and stimulation within a certain duration and intensity range elicits after-effects, which can last from 90 min to more than 24 h (Nitsche and Paulus, 2001; Monte-Silva et al., 2013; Agboada et al., 2020). Even though physiological mechanisms of tDCS-induced plasticity are not yet fully understood, it is assumed that its effects are based on long-term potentiation–(LTP) and long-term depression-like (LTD) mechanisms, with primarily glutamatergic processes involved, and that GABA modulation has a gating function on respective plasticity (Stagg and Nitsche, 2011; Yavari et al., 2018). tDCS has a potential to modulate cognitive, motor, perceptual and emotional processes, based on respective physiological alterations (Yavari et al., 2018).

rTMS is another non-invasive brain stimulation method for studying neuroplasticity and modulating cortical excitability (Pascual-Leone et al., 1998; Hallett, 2007). Unlike tDCS, rTMS uses magnetic fields to induce electrical discharges of respective target areas of the brain. Trains of magnetic pulses at varying frequencies are delivered via a coil positioned on the scalp. In general, low frequency stimulation (</=1 Hz) has inhibitory effects, while high frequency stimulation (>5 Hz) results in excitatory effects (Klomjai et al., 2015). Similar to tDCS, it is assumed that rTMS after-effects are based on LTP- and LTD-like mechanisms of synaptic plasticity (Hallett, 2007; Lefaucheur et al., 2014). Deep TMS is similar to conventional rTMS, but stimuli are conducted via an H-coil, which is suggested to enable stimulation of deeper cerebral regions (Levkovitz et al., 2015). Theta burst stimulation (iTBS) is a form of patterned rTMS with stimulation delivered in repetitive bursts of 50 Hz five times per second, which delivers similar plasticity responses as rTMS, but via shorter stimulation protocols (Huang et al., 2005; Di Lazzaro et al., 2011; Bakker et al., 2015). Due to improved knowledge of the neural circuits outlined above, the availability of interventions to tackle respective circuits in humans, and considering the need for adjunctive treatment protocols, NIBS methods are attractive candidates for modulating fear and extinction memory. Here we review studies related to fear and extinction memory and NIBS in clinical and non-clinical samples, as well as in human and animal models, to give an overview about the state of the art, and derive hypotheses about future developments in this field, with respect to optimization and mechanisms involved.

## Methods

### Inclusion and Exclusion Criteria

To select papers with sound quality, only peer-reviewed published papers were included in this review. Inclusion criteria were: (1) research articles in healthy and clinical samples, as well as research in animal models published in English language; (2) research articles based on fear memory and extinction (including studies that used exposure therapy protocols), which employed non-invasive brain stimulation methods (i.e., tDCS, and TMS); (3) research articles with a sufficiently detailed description of respective intervention protocols (e.g., duration, intensity/frequency, experimental design), and also case studies. Exclusion criteria: (1) research articles in other than English language; (2) research articles that are not based on fear extinction and exposure protocols in combination with NIBS methods; (3) review articles, abstracts and commentaries.

### Search Strategy, Information Sources, and Study Selection

**Figure 1:**
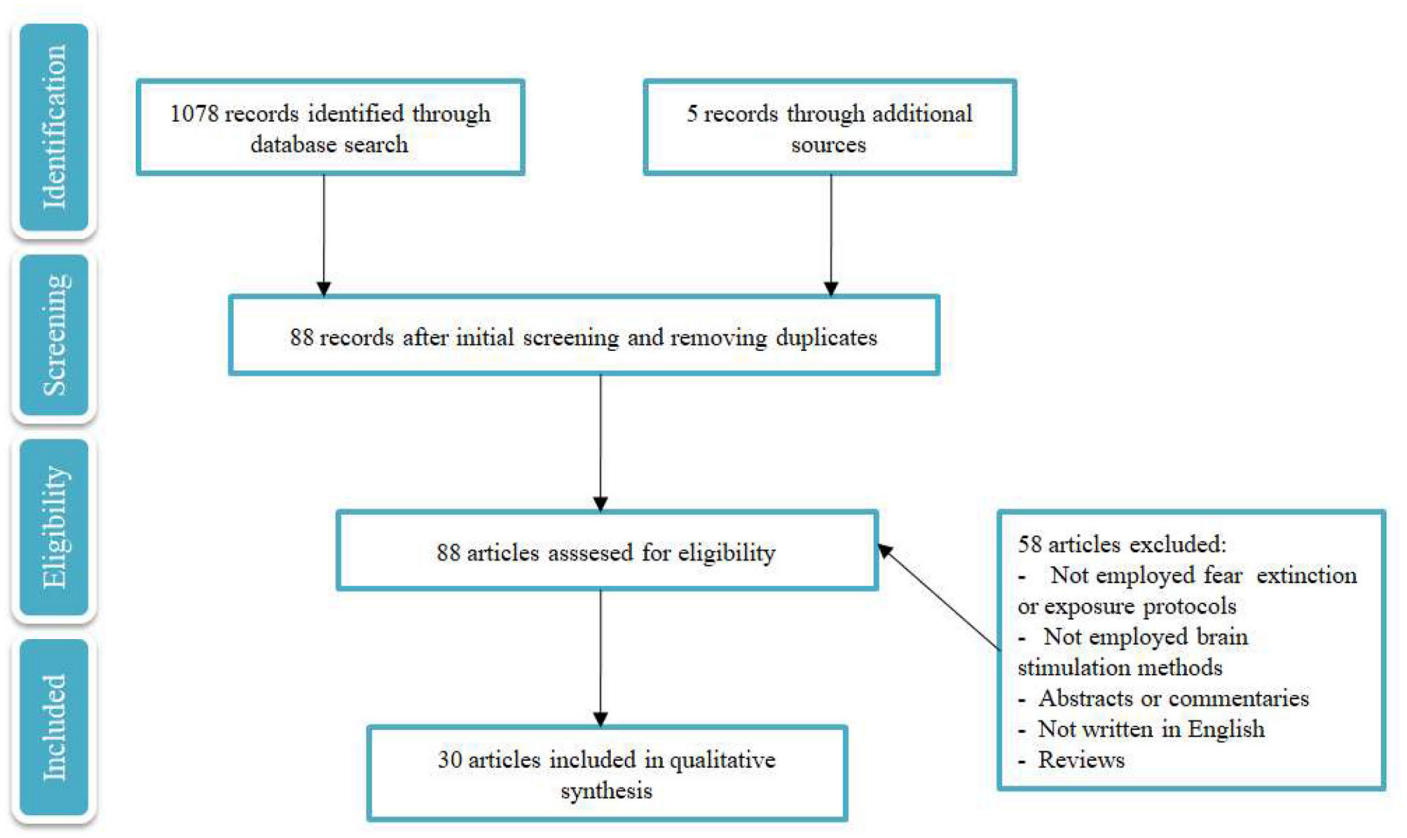
PRISMA flow diagram selected for qualitative analysis of the respective studies.

The procedure was conducted by one of the authors (VM) in accordance with the guidelines of Preferred reporting items for systematic reviews and meta-analyses (PRISMA) (Moher et al., 2009) (for details, see Figure 1). An extensive search was conducted via PubMed and Google Scholar databases. Key words used in the search were: “transcranial direct current stimulation” OR “transcranial magnetic stimulation” AND “fear extinction” OR “fear memory” OR “exposure therapy,” which led to six pairs of key words. Initially, 1,078 records were identified. After screening and removing duplicates, 88 studies remained eligible for the review. “Transcranial direct current stimulation” and “fear memory” led to eight results (six of these were excluded; four did not use fear conditioning/extinction or exposure protocols, and two were reviews). “Transcranial magnetic stimulation” and “fear memory” led to four results, three studies were excluded because they were not based on fear conditioning/extinction or exposure protocols, and one was a review; “transcranial direct current stimulation” and “fear extinction” led to 33 results (21 of these were excluded; six did not use fear extinction or exposure protocols, two did not use brain stimulation methods, two of these were abstracts, two were commentaries, one was not written in English, and eight were reviews), “transcranial magnetic stimulation” and “fear extinction” lead to 22 results (14 of these were excluded; two were abstracts, eight did not use fear conditioning/extinction or exposure protocols, and four were reviews), “transcranial direct current stimulation” and “exposure therapy” led to five results (four were excluded; one did not use fear conditioning/extinction or exposure protocols, one did not use brain stimulation methods and two were reviews). “Transcranial magnetic stimulation” and “exposure therapy” led to 15 results (13 were excluded; five studies did not use fear conditioning/extinction or exposure protocols, four were reviews, three did not use brain stimulation methods, and one was an abstract). Five more studies were identified through other sources (i.e., research articles) resulting in 30 studies included in this review. Research of the records was conducted until 1st of March 2020. In addition, a study by Ney et al. (2021) was published recently and included in the review.

### Outcome Variables

Major outcome variables in fear memory studies (both tDCS and TMS studies) were skin-conductance response (SCR), self-reported fear, anxiety and stress inventories, visual analog scales and approach-avoidance tasks (see Table 1 for more details). In animal studies, fear memory (short-term and long-term contextual memory, auditory fear memory) was measured by freezing and latency to freezing behaviors (see Table 2). In fear extinction studies conducted in healthy humans (both tDCS and TMS studies), respective outcome measures were again SCR, fear potentiated startle, approach-avoidance tasks, and self-report scales of fear. In the clinical population, outcome variables were most frequently the symptoms measured by self-report scales and inventories of respective disorders (PTSD, OCD, specific phobia) (see Tables 1, 3, for details). In animal studies, fear extinction was measured similarly to fear memory by freezing and latency to freezing as well as the sensitized fear test and object recognition task (see Table 4 for details).

**Table 1:** Effect of tDCS on fear memory and fear extinction in healthy and clinical groups.

**References**	**Study type**	**N/Groups**	**Gender M/F (Mean age ± SD)**	**Target area**	**Target/return electrode position**	**Polarity**	**Size**	**Online/offline stimulation**	**Intensity/duration**	**Type of CS/US**	**Reinforcement rate**	**Outcome measures**	**Outcome direction**
**FEAR MEMORY**													
Asthana et al. (2013)	RCT, sham controlled, single blind	49 healthy participants/anodal, cathodal and sham	24/25 (22.58 ± 2.24)	Left dlPFC	F3/left mastoid	Anodal/cathodal	35 cm^2^	Offline, after acquisition and break (10–20 min)	1 mA/12 min	Colored blue and yellow squares/Scream	75%	SCR	Diminution of fear memory
Mungee et al. (2014)	RCT, sham controlled, single blind	50 healthy participants/active and sham tDCSgroups	28/22 (N.R.)	Right dlPFC/vmPFC	F4/contralateral supraorbital area	Anodal	15 cm^2^	Offline, on day 2 immediately after reminding	1 mA/20 min	Blue and yellow squares/Electrical shock	38%	SCR	- Enhancement of fear memory
Mungee et al. (2016)	RCT, sham controlled, single blind	17 healthy participants/active and sham tDCS groups	5/12 (N.R.)	Right dlPFC/vmPFC	F4/contralateral supraorbital area	Cathodal	15 cm^2^	Offline, on day 2 immediately after reminding,	1 mA/20 min	Blue and yellow squares/Electrical shock	38%	SCR	- No effect on fear memory
**FEAR EXTINCTION**													
Abend et al. (2016)	RCT, sham controlled, double blind	45 healthy participants/tDCS, tACS and sham groups	24/21 (25.2 ± 5.7)	mPFC	Between Fpz and Fp1/occipital bone (Oz)	Anodal	35 cm^2^	Online, during extinction	1.5 mA/20 min	Two female faces/Scream	80%	SCR, self-reported fear	- Anodal tDCS led to overgeneralization
van't Wout et al. (2016)	RCT, sham controlled, single blind	44 healthy participants/active and sham tDCS groups	23/21 (27.34 ± 8.18)	Left vmPFC	AF3/contralateral mastoid	Anodal	15 cm^2^	Online, started before and continued during extinction	2 mA/10 min	Red, blue and yellow lights/Electrical shock	60%	SCR	- tDCS during the first extinction block enhanced late extinction of the second extinction block
Dittert et al. (2018)	RCT, sham controlled, double blind	84 healthy participants/two active and two sham tDCS groups	38/46 (24.25 ± 4.07)	Right and left vmPFC	Electrodes positions: M20, M21, I20, I21, J13, J14 for the left and M9, M10, I9, I10, J6, J7 for the right electrode pad (slightly below F7 and F8)	Anodal	16 cm^2^	Online, started during the break between acquisition and extinction and lasted until the end of the extinction	1.5 mA/20 min	Two female faces/Scream	80%	SCR, self-report measures (valence, arousal and CS-US contingency STAI-X1, PANAS)	-Enhancement of early extinction - No differential effect of current flow direction on early extinction - left anodal tDCS reduced state anxiety
Ganho-Ávila et al. (2019)	RCT, sham controlled, single blind	41 healthy participants/cathodal and sham tDCS groups	0/41 (20.42 ± 4.99)	Right dlPFC	F4/contralateral deltoid	Cathodal	24.75cm^2^	Offline, on day 2, after verbal recall of CS+ color	1 mA/20 min	Blue and yellow squares/Scream	75%	SCR, self-report measures (valence, arousal, contingency and expectancy), STAI-S, AAT.	- No short-term effect on fear extinction - Enhancement of fear memory retention - Enhancement of stimuli discrimination
Vicario et al. (2020b)	RCT, sham controlled, single blind	23 healthy participants/anodal and sham tDCS groups	10/13 (24.15 ± 4.92)	Left vmPFC	AF3/contralateral mastoid	Anodal	25 cm^2^	Online, during extinction	2 mA/20 min	Colored circles/Electrical shock	71%	SCR	-Enhanced fear extinction and recall
Ney et al. (2021)	RCT, sham controlled, single blind	30 healthy participants/anodal and sham tDCS groups	10/20 (24.60 ± 7.30)	Left vmPFC	AF3/contralateral mastoid	Anodal	25 cm^2^	Offline, after extinction	2 mA/10 min	Colored circles/Electrical shock	62.5%	SCR	- Anodal tDCS impaired fear extinction retention on day 2
**CLINICAL GROUPS**													
van't Wout et al. (2017)	RCT, sham controlled Blinding mode not reported	28 PTSD/two groups (stimulation during extinction learning vs after extinction)	28/0 (56.25 ± 12.3)	Left vmPFC	AF3/contralateral mastoid process	Anodal	25 cm^2^	Online and offline, during and after extinction	2 mA/10 min (single-session)	Red, blue and yellow light./Electrical shock	60%	SCR	- Enhanced early fear extinction recall when tDCS was applied after extinction
Todder et al. (2018)	RCT, within-subject sham controlled, double blind	12 refractory OCD patients/one group	7/5 (38.5 ± 12)	mPFC	Fpz/right shoulder	Anodal/cathodal	35 mm^2^	Offline, after exposure and between presentation of obsession provoking stimuli	2 mA/20 min (3 sessions for each polarity)	Individualized anxiety provoking stimuli	N/A	7-point VAS, Y-BOCS, HAMA, MADRS	- Reduction of obsession-induced anxiety after cathodal stimulation
van't Wout-Frank et al. (2019)	RCT, sham controlled, single blind	12 PTSD patients	12/0 (40.5 ± 8.8)	Left vmPFC	AF3/right posterior occipital (PO8)	Anodal	25 cm^2^	tDCS started simultaneously with VR, online	2 mA/25 min (6 sessions)	N/A	N/A	SCR, self-reported PTSD symptoms	- Greater decrease in SCR in real tDCS group - RealtDCS group continued to improve during the 1-month follow-up.

*DlPFC, dorsolateral prefrontal cortex; Mpfc, medial prefrontal cortex; vmPFC, ventromedial prefrontal cortex; VR, virtual reality; OCD, Obsessive-Compulsive disorder; PTSD, Posttraumatic stress disorder; SCR, Skin conductance response; STAI-X1, State-trait anxiety inventory-state form; PANAS, Positive and Negative Affect Schedule; VAS, visual analog scale; AAT, approach avoidance task; MADRS, Montgomery-Åsberg Depression Rating Scale; Y-BOCS, Yale-Brown Obsessive-Compulsive Scale; HAMA, Hamilton Anxiety Rating Scale; N/A = not applicable*.

**Table 2:** Effects of tDCS on fear memory in animal models.

**References**	**Study type**	**Subjects and groups**	**Target area**	**Target electrode position**	**Return electrode position**	**Stimulation polarity**	**Size of target and return electrodes**	**Online/offline stimulation**	**Intensity (mA)**	**Duration (minutes)**	**Drug/doses**	**CS/UCS**	**Outcome measures**	**Outcome**
Manteghi et al. (2017)	RCT, placebo and sham controlled	64 male NMRI mice, 8 groups	Prefrontal region(right)	1 mm anterior and 1 mm right to the Bregma	Chest	Anodal	3.5 mm^2^, 9.5 cm^2^	Offline, 24 h before conditioning	0.2	20	ACPA/0.01, 0.05, and 0.1 mg/kg	Tone/foot shock	- Freezing duration and latency - Grooming and rearing duration	- tDCS improved short-term contextual fear memory (0.01 and 0.05 doses of ACPA) and long-term contextual and auditory fear memory formation (all doses of ACPA)
Abbasi et al. (2017)	RCT, sham controlled	41 male NMRI mice, 5 groups	Prefrontal region(left)	1 mm anterior and 1 mm left to the Bregma	Chest	Anodal, cathodal	3.5 mm^2^, 9.5 cm^2^	Offline, immediately before fear conditioning	0.2	20 and 30	No drugs	Tone/foot shock	Freezing duration and latency	- tDCS impaired acquisition of contextual and cued fear memory (Contextual: 20 and 30 min of anodal, 30 min cathodal; Cued: 20 min anodal, 30 min cathodal)
Nasehi et al. (2017b)	RCT, placebo and sham controlled	120 male NMRI mice, 9 groups	Prefrontal region (right)	1 mm anterior and 1 mm right to the Bregma	Chest	Anodal, cathodal	3.5 mm^2^, 9.5 cm^2^	Offline, 1 day before/immediately after fear conditioning	0.2	20	Propranolol/0.1 mg/kg	Tone/foot shock	Freezing duration and latency	- Post-training cathodal stimulation itself facilitated contextual and auditory fear memory retrieval - Pre-training application of cathodal tDCS combined with pre- or post- training propranolol restored auditory fear memory retrieval - Pre- and post-cathodal tDCS in combination with pre-training propanolol increased fear memory retrieval and combined with post-training propanolol increased contextual fear memory - Pre- or post-training anodal tDCS in combination with pre-training propranolol increased contextual and reversed auditory fear memory retrieval - Pre-training anodal combined with post-training propranolol increased contextual fear memory retrieval
Nasehi et al. (2017a)	RCT, placebo and sham controlled	120 male NMRI mice, 9 groups	Prefrontal cortex (left)	1 mm anterior and 1 mm left to the Bregma	Chest	Anodal, cathodal	3.5 mm^2^, 9.5 cm^2^	Offline, 1 day before/immediately after fear conditioning	0.2	20	Propranolol/0.1 mg/kg	Tone/foot shock	Freezing duration and latency	- Pre-training cathodaltDCS itself increased contextual fear memory retrieval - Pre- and post-training cathodal tDCS in combination with propanolol pre-training increased fear memory retrieval - Pre- and post-training cathodal tDCSwith post-training propanolol increased contextual fear memory - Pre- and post-training anodal tDCS with pre-training propanolol increased contextual memory retrieval - Pre-training anodal tDCS with pre-training propranolol increased auditory fear memory retrieval

**Table 3:** Effect of rTMS on fear extinction in clinical groups.

**References**	**Study type**	**N/Groups**	**Syndrome**	**Gender, M/F (age, Mean ± SD)**	**Target area**	**Coil position**	**Online/offline stimulation**	**Pulses per session/duration**	**Frequency/Intensity/Coil shape**	**Outcome measures**	**Outcome direction**
Notzon et al. (2015)	RCT, Single blind, active (control site) and sham controlled	83/4	Spider phobia	1) 4/37 (27.51 ± 9.4) 2) 5/37 (25.43 ± 7.37) 3) 4/36 (25.85 ± 7.65) 4) 5/38 (27.02 ± 9.23)	Left dlPFC	F3	Offline, before the VR challenge	iTBS/600/3 min	15 Hz/80% RMT/figure of 8	FSQ, SPQ, ASI, psychophysiological measures (HR, HRV, SCR)	iTBS - had no general effect of on anxiety, disgust, HR and SCR. - significantly increased sympathetic activity
Herrmann et al. (2017)	RCT, Double blind, sham controlled	39/2	Acrophobia	1) 6/13 (46.6 ± 13.7) 2) 7/13 13/26 (43.2 ± 12.6)	mPFC	Fpz	Offline, before exposure	rTMS/1560/2 × 20 minutes	10 Hz/100% RMT/Round	AQ, BAT	- rTMS reduced phobic anxiety immediately after two sessions of VR exposure therapy. - No differences between active and sham rTMS stimulation at follow up.
Osuch et al. (2009)	Double-blind, sham controlled	9/1	PTSD	1/8 (41.4 ± 12.3)	Right dlPFC	5 cm rostral to APB muscle hotspot	Online, during exposure to emotionally provoking memories.	rTMS/1800/30 min per session/20 sessions	1 Hz/100% RMT/figure of 8	CAPS, IES, HDRS	- Active rTMS showed a larger improvement of hyperarousal symptoms compared to sham
Isserles et al. (2013)	RCT, Double-blind, sham controlled, controlled for traumatic event as well	26/3	PTSD	1) 7/2 (49 ± 12.5) 2) 8/1 (40.4 ± 10.5) 3) 5/3 (40.5 ± 9.8)	mPFC	H-Coil designed tostimulate the mPFC.	Offline, after exposure to the traumatic event	Deep rTMS/1680/15.5 min per session/12 sessions	20 Hz/120% RMT/H-coil	CAPS, PSS-SR, HDRS, BDI, psychophysiological data (HR)	-Symptom improvement by dTMS (revealed by changes in CAPS, PSS-SR, HDRS, BDI and HR)
Fryml et al. (2019)	RCT, Double blind, sham controlled	8/2	PTSD	1) 2/1 (30 ± 2.6) 2) 5/0 (27 ± 2.1)	Leftor right dlPFC	6 cm anterior to the right hand motor thumb area	Online, duringprolonged exposure therapy	rTMS/6000/30 min per session/8 sessions	10Hz/120% RMT/figure of 8	CAPS, HRSD	- Change in HRSD showed antidepressant benefit of rTMS. - CAPS scores showed no significant improvement
Carmi et al. (2018)	RCT, Double blind, sham controlled	41/3	OCD	1) 9/7 (36 ± 2.1) 2) 4/4 (28 ± 3.1) 3) 7/7 (35 ± 3.5)	mPFCand ACC	4 cm anterior to theleg motor spot at midline	Offline, following symptom provocation	Deep rTMS/HF: 2000 LF: 900/25 sessions	HF: 20 Hz, LF: 1 Hz/HF: 100% RMT, LF: 110% RMT/H7 Coil	YBOCS, CGI-I	- Symptoms improved by high frequency deep rTMS (YBOCS, CGI-I)
Carmi et al. (2019)	RCT, Double blind, sham controlled	94/2	OCD	1) 20/27; (41.1 ± 11.97) 2) 19/28 (36.5 ± 11.38)	mPFCand ACC	4 cm anterior to the foot motor spot	Offline, following symptom provocation	Deep rTMS/2,000/29 sessions	20 Hz/100% RMT/H7 coil	YBOCS, CGI-I, CGI-S, and Sheehan Disability Scale scores	- Symptom improvement by dTMS (YBOCS, CGI-I, CGI-S)
Adams et al. (2014)	Case study, Single blind	1	OCD	1/0 (52 yo)	Pre-supplementary motor area	50% of the distance between the Fz and FCz	Offline, immediately prior ERP exercises	rTMS/1200/20 min per session/15 sessions	1 Hz/110% RMT/figure of 8	YBOCS,PHQ-9,GAD-7, DOCS	- Symptom improvement in YBOCS, DOCS, GAD-7, and PHQ-9
Grassi et al. (2015)	Case study	1	OCD	0/1 (32 yo)	Left dlPFC	N.R.	Offline, immediately prior ERP exercises.	rTMS/1800/N.A./10 sessions	10 Hz/80% RMT/NR	Y-BOCS, CGI-I, HAM-D, GAF	- Symptom improvement in Y-BOCS, CGI-I, GAF

*ACC, Anterior cingulate cortex; dlPFC, dorsolateral prefrontal cortex; mPFC, medial prefrontal cortex; vmPFC, ventromedial prefrontal cortex; iTBS, intermittent theta burst stimulation; rTMS, repetitive transcranial magnetic stimulation; LF, low frequency; HF, high frequency; MEP, motor evoked potential; RMT, resting motor threshold; PTSD, Posttraumatic stress disorder; OCD, Obsessive-Compulsive disorder; HR, heart rate; HRV, heart rate variability; SCR, Skin conductance response; EEG, electroencephalography; FPS, Fear potentiated startle; fNIRS, Functional near-infrared spectroscopy; CAPS, Clinician Administered PTSD Scale; IES, The Impact of Event Scale; SPQ, Spider Phobia Questionnaire; FSQ, Fear of Spiders Questionnaire; ASI, Anxiety Sensitivity Index; AQ, acrophobia questionnaire; BAT, Behavioral Avoidance Test; BDI, Beck Depression Inventory; HDS, Hamilton Depression scale; HDRS, Hamilton Rating Scale for Depression; PGI-T, Patient Global Impression of Improvement; Y-BOCS, Yale-Brown Obsessive-Compulsive Scale; GAF, Global Assessment of Functioning; GAD-7, General Anxiety Disorder Scale; PHQ-9, Patient Health Questionnaire; DOCS, Dimensional Obsessive-Compulsive Scale; CGI-S, Clinical Global Impression—severity scale; CGI-I, The CGI—improvement scale; ERP, Exposure Response Prevention; N/A, not applicable; N.R., not reported*.

**Table 4:** Effect of rTMS on extinction in animal models.

**References**	**Study type**	**Subjects and groups**	**Target area**	**Coil position**	**Online/offline stimulation**	**Pulses per session/duration**	**Frequency/Intensity/coil shape**	**Drug/doses**	**Reinforcement rate**	**CS/US**	**Outcome measure**	**Outcome direction**
Baek et al. (2012)	RCT, sham controlled	35 rats, 2 experiments, active and sham group in each experiment;	Infralimbic cortex	3 mm anterior to bregma	Offline and online, rTMS was finished either 5 min before or applied during extinction	1,000 pulses/10 min	10 Hz/90% MT/Modified figure-of-eight coil,	None	100%	Sound/Foot shock	Freezing duration	- rTMS paired with CS significantly facilitated fear extinction
Legrand et al. (2019)	RCT, sham and vehicle controlled study	140 mice, 8 groups	Infralimbic cortex	2 mm anterior to the bregma	Offline, from day 7 to 12, five rTMS sessions or sham sessions were applied 24 h apart	750 pulses/7 min and 48 s × 5 sessions	12 Hz/115% MT/Circular coil	Fluoxetine/15 mg/kg	N/A	Chamber/Foot shock	- Freezing duration and latency - Performance in object recognition task - c-Fos neuronal expression	rTMS - enhanced fear extinction. - reversed short-term memory impairments. - evoked c-Fos activity in the vmPFC (infralimbic cortex), the basolateral amygdala and the ventral CA1

## Results

### Effects of tDCS on Fear Memory

#### Effects of tDCS on Fear Memory in Animal Models

Four studies applied tDCS in animal models with the aim to modulate fear memory (Table 2). Abbasi et al. (2017) performed a study in mice that received anodal, or cathodal tDCS for 20 or 30 min at an intensity of 0.2 mA, or sham stimulation over the left prefrontal cortex. tDCS was delivered a few minutes before fear conditioning with an electrical shock. Twenty-four hours later, animals were tested in a contextual fear memory test (absence of CS and US, but context of fear conditioning), and a cued fear memory test (different context, CS presented). Several measures of anxiety-like behaviors were assessed (i.e., latency to freezing, duration of freezing, locomotor activity). Anodal and cathodal tDCS impaired acquisition of contextual and cued fear memory, largely independent from the respective stimulation duration (Table 2).

In three other studies, the impact of priming tDCS 1 day before fear conditioning or application of tDCS after fear conditioning was explored, with a specific dedication to the re-establishment of fear memory compromised by pharmacological interventions. Manteghi et al. (2017) applied anodal or sham tDCS in combination with the cannabinoid receptor agonist arachidonylcyclopropylamide (ACPA; 0.01, 0.05, 0.1 mg/kg, or vehicle). ACPA was injected 15 min before fear conditioning to impair fear learning and memory (Nasehi et al., 2016). tDCS was delivered over the right frontal region (i.e., 1 mm anterior and 1 mm to the right from the Bregma) for 20 min at an intensity of 0.2 mA 1 day before auditory fear conditioning. Twenty-four hours, and 2 weeks after training, animals were tested with a contextual associative memory test (i.e., same conditioning context, but without US and CS) and an auditory associative memory test (i.e., different context than training, but exposed to the CS). tDCS selectively improved drug-induced impairments of short-term contextual fear memory, but it did not affect short-term contextual and auditory memory in the absence of ACPA. After 14 days, tDCS restituted all memory functions which were compromised by ACPA. Nasehi et al. (2017b) tested the influence of tDCS on fear memory on mice. Animals received anodal, cathodal, or sham tDCS for 20 min over the right frontal cortex at an intensity of 0.2 mA 1 day before or immediately post-training (fear conditioning), without or with pre- and post-training administration of propranolol, which reduces fear memory (Lonergan et al., 2013). On the next day, animals were tested for contextual fear memory and 1 h later for auditory fear memory. When propranolol was applied prior to the training, and anodal stimulation prior or after the training, contextual fear memory retrieval increased, and the drug-induced impairment of auditory fear memory was reversed. Moreover, when stimulation was applied prior to the training and propranolol was administered after training, a selective improvement of contextual, but not of auditory fear memory retrieval was observed. Cathodal stimulation abolished the effects of propranolol on auditory fear memory only when performed prior to the training.

Using an otherwise identical experimental design, Nasehi et al. (2017a) applied tDCS over the left frontal cortex. The main result shows that pre- or post-training anodal tDCS applied when propranolol was administered prior to training reversed the effect of propranolol on contextual fear memory acquisition (Nasehi et al., 2017a). Moreover, regardless of the specific timing of cathodal stimulation, and administration of propranolol, stimulation re-established the propranolol-induced diminution of contextual memory retrieval.

Overall, the results of these studies show that tDCS can alter fear memories. However, specific effects are heterogeneous. Prefrontal stimulation immediately before fear acquisition reduced, whereas post-training cathodal enhanced fear memory. Moreover, tDCS applied 1 day before fear acquisition restituted pharmacologically compromised fear memory. An important limitation of the examined literature is that, in most cases, no sufficient information is reported about which specific portion of the prefrontal cortex was stimulated. This makes it problematic to provide mechanistic explanations of the available data. Other potential limitations of the respective studies, which make interpretation of the data difficult, are related to the adopted protocols, which could have induced metaplastic effects. Finally, the absence of online stimulation studies, which would probably have provided fewer complex effects, and rTMS studies for comparison with the currently available tDCS literature, is another limitation.

#### Effects of tDCS on Fear Memory in Healthy Humans

The database research identified 3 tDCS studies performed in healthy humans that aimed to affect fear memory (Table 5). Asthana et al., 2013 performed a study to investigate the effect of anodal and cathodal tDCS on fear memory consolidation. Participants received anodal, cathodal or sham tDCS over the left dlPFC (return electrode over left mastoid) with 1 mA for 12 min in connection with a 2-day fear conditioning protocol. During the first day, participants went through the habituation and fear acquisition phase. Colored circles were used as CS and a scream as US. Stimulation was started 10 to 20 min after the acquisition phase. On the second day, participants were again exposed to the CS without US to assess consolidation of fear memory. Fear conditioning was assessed by SCR. Cathodal stimulation resulted in significantly lower SCR values compared to anodal and sham, suggesting a role of the left dlPFC in fear memory consolidation. Mungee et al. (2014) aimed to assess the effects of tDCS on fear memory reconsolidation in a 3-day protocol. Fear acquisition was performed on the first day with colored squares as CS and an electrical shock as US. On the second day, participants were first reminded of the CS+ (presentation of one CS+ that was combined with electrical shock on day 1) and stimulated with tDCS immediately afterwards for 20 min with an intensity of 1 mA, with the anode placed over the right dlPFC, and the cathode over the left supraorbital area. Assessment of fear memory was performed on the third day via presentation of the CS stimuli without US. Fear memory was assessed with SCR. The results showed an enhancement of fear memory by tDCS, suggesting a role of the right dlPFC and/or left vmPFC in fear memory reconsolidation. In a second study of the same group (Mungee et al., 2016), the participants performed the same protocol as in the previous study, but with reversed electrode positions for tDCS (i.e., right dorsolateral prefrontal–cathodal, left supraorbital–anodal). The results showed no change in SCR.

**Table 5:** Effect of rTMS on fear memory and extinction in healthy participants.

**References**	**Study type**	**N/Groups**	**Gender, M/F (age, Mean ± SD)**	**Target area**	**Coil position**	**Online/offline stimulation**	**Pulses per session/duration**	**Frequency/Intensity/Coil shape**	**Type of CS/US**	**Reinforcement rate**	**Outcome measures**	**Outcome direction**
Borgomaneri et al. (2020)	RCT, Single-blind, active (control site) and sham controlled	84/6	1.6/8 (23.9 ± 2.3) 5/9 (23.1 ± 2.6) 3/11 (21.6 ± 2.0) 8/6 (22.4 ± 3.7) 6/8 (23.2 ± 1.8) 6. 5/9 (24.4 ± 3.1)	Left and right dlPFC	F3 and F4	Online, during reconsolidation of fear memory	900/15 min	1 Hz/110% RMT/figure of 8	Room pictures/electrical shocks	60%	SCR, contingency ratings	Both l- and r- dlPFCrTMS -diminished expression of fear response - prevented return of fear response
Guhn et al. (2014)	RCT, Single-blind, sham controlled	85/2	Active group: 21/19 (23.9 ± 3.0); Sham: 22/23 (24.6 ± 4.5)	mPFC	Fpz	Offline, between acquisition and extinction	1,560/20 min	10 Hz/110% RMT/Round	Two male faces/scream	50%	SCR, FPS, fNIRS, and self-report scales	rTMS - enhanced fear extinction learning - Improved extinction recall
Raij et al. (2018)	Single-blind, active (control site) controlled	28/2	23/5 (28yo;19-51)	vmPFC	Left posterior PFCwith strong or weak vmPFC connectivity	Online, during extinction	28/4 trains, 7 pulses per train	20 Hz/100% RMT/figure of 8	Red, blue and yellow lights/Electrical shocks	62.5%	SCR	rTMS enhanced fear extinction recall

Although preliminary, tDCS appears to modulate fear memory in human subjects. Specifically, cathodal stimulation of the left dlPFC led to disruption of fear memory consolidation, while anodal stimulation of the right dlPFC (which however might have also involved effects of cathodal tDCS over left mesio-frontal areas) enhanced fear memory retrieval. Furthermore, the mentioned studies targeted different memory consolidation processes, i.e., consolidation (Asthana et al., 2013) vs. reconsolidation (Mungee et al., 2014, 2016).

#### Effects of rTMS on Fear Memory in Healthy Humans

Only one recently published study has tested the effects of low-frequency excitability-diminishing rTMS (1 Hz, 110% RMT, stimulation duration 15 min) on fear memory in healthy humans (Borgomaneri et al., 2020) by targeting, in separate groups, the left or right dlPFC in a 3-day—sham controlled–protocol. During the first day, participants conducted a fear conditioning task [two pictures of a room (CS), one paired with an electrical shock (US)]. Twenty-four hours afterwards, fear memory reactivation was induced via a reminder cue (two times presentation of the CS+ without US), and then rTMS was conducted. On day 3, memory recall, extinction and reinstatement measures were performed. Compared to the sham rTMS group, participants of the left and right dlPFCrTMS groups exhibited decreased physiological expression of fear in the memory recall test (i.e., reduced SCR), only when rTMS was administered within the reconsolidation time window (i.e., 10 min after the exposure to a reminder cue that reactivated a fear memory acquired 1 day before). Moreover, dlPFC-rTMS prevented subsequent return of fear after extinction training.

Since no effects were reported in participants tested immediately after dlPFC-rTMS or dlPFC-rTMS without preceding fear-memory reactivation, the authors suggest a specific role of dlPFC in fear-memory reconsolidation. Overall, this result is in line with previous evidence from tDCS studies (Mungee et al., 2014) documenting a modulation of fear memory with anodal tDCS of the right dlPFC applied in the context of fear memory reconsolidation.

### Effects of NIBS Methods on Fear Extinction

#### Effects of rTMS on Fear Extinction in Animal Models

Two studies were identified that fulfilled the inclusion criteria (Table 4). Baek et al. (2012) conducted a 3-day protocol in rats to assess the effect of excitability-enhancing rTMS on fear extinction with real or sham stimulation applied before or during extinction. The coil was positioned over the PFC (~3 mm anterior to Bregma, in a region that would be able to target vmPFC, according to the authors aim) and stimulation was applied for 10 min at 10 Hz frequency. On the first day, rats were exposed to auditory stimuli for habituation, and then exposed to auditory stimuli (CS) paired with a foot shock (US). The next day, in experiment 1, rTMS or sham stimulation were finished 5 min before the extinction process. In experiment 2, stimulation was applied simultaneously with the extinction protocol. On day 3, the CS was presented without the US to assess fear extinction memory. Freezing behavior served as dependent variable. Rats who received rTMS during, but not before extinction showed significantly less freezing behavior than the sham group, during, and 1 day after extinction. Legrand et al. (2019) conducted a study in an experimental mouse PTSD model to assess the effect of rTMS on fear extinction and related neurocircuits. The mice were split into non-stressed and stressed (PTSD) groups, and received sham or real rTMS, combined with the serotonin reuptake inhibitor fluoxetine, which is used for PTSD treatment (Ariel et al., 2017), or vehicle. Facilitatory 12 Hz rTMS was applied over the vmPFC (the coil was positioned latero-medial to promote bilateral effects) for 7 min and 48 s per session. On the first day, the PTSD mice were exposed to foot shocks to induce stress-like effects. From the second day on, a treatment with fluoxetine or vehicle was conducted. From day 7 to 12, five rTMS or sham stimulation sessions were applied. At day 17 and 18, the mice underwent object recognition tasks and at day 22, the mice were re-exposed to the conditioning chamber. Object recognition task (ORT) performance, duration of freezing and latency to freezing were used as indicators of anxiety-like behaviors. PTSD rTMS mice explored novel objects significantly more than the PTSD sham group and showed a decreased duration of freezing. rTMS furthermore increased c-Fos activity in the infralimbic cortex, basolateral amygdala and the ventral CA1. Taken together, results suggest that rTMS enhanced fear extinction and reversed short-term memory impairments, which was associated with early gene expression in extinction-related areas. In summary, these studies suggest that application of facilitatory rTMS over the PFC can influence fear extinction via modulation of specific brain circuits involved in extinction, such as the IL, amygdala, and hippocampus.

#### Effects of rTMS on Fear Extinction in Healthy Humans

Two studies related to the application of rTMS to influence fear extinction were identified (Table 5). Guhn et al. (2014) conducted a study to assess the influence of high frequency rTMS on fear extinction in a 2-day sham-controlled protocol. Stimulation was delivered with 10 Hz frequency (110% RMT) for 20 min over the bilateral mPFC. On the first day, participants were familiarized with the stimuli (habituation), and subsequently fear acquisition took place. Human faces served as CS, and a scream as US. rTMS was applied immediately before extinction. On the second day, extinction recall was assessed. SCR, fear potentiated startle (FPS), functional near-infrared spectroscopy (fNIRS), and subjective ratings were obtained to assess fear responses. Active rTMS enhanced fear extinction, as measured by FPS, SCR, and subjective valence and arousal ratings. Furthermore, the active rTMS group showed significantly reduced FPS magnitudes during extinction recall. Raij et al. (2018) assessed effects of rTMS on fear extinction in healthy subjects in a 3-day protocol. Stimulation (20 Hz, 100% RMT) was applied over two spots of the PFC with strong or weak connections with the vmPFC, as revealed by fMRI. On the first day, participants were conditioned to two colors (CS+) associated with an electrical shock (US), while another color (CS-) was not paired with the electrical shock. On the second day, the extinction protocol was applied with only one CS+ paired with online rTMS. rTMS was applied four times for 300 ms after each CS+. SCR during extinction recall was significantly reduced when the cue was paired with rTMS over the area that exhibited strong functional connectivity with the vmPFC.

Although preliminary, these results show a potential of high frequency rTMS to enhance fear extinction and recall when applied over the mPFC, and areas which are strongly connected with the vmPFC.

#### Effects of tDCS on Fear Extinction in Healthy Humans

Five studies were identified that meet the inclusion criteria. Four of these studies applied tDCS over the mPFC or vmPFC, and one over the dlPFC (Table 3). Abend et al. (2016) assessed the effect of tDCS on fear extinction and recall. Participants received anodal tDCS or sham stimulation over the mPFC (return electrode over occipital bone) for 20 min with a constant current of 1.5 mA in a 3-day protocol. On the first day, participants underwent classical conditioning, where one of two female faces (CS) was combined with a scream (US). During extinction on the second day, the CS was presented without the US, except for the first CS+ trial, which was reinforced as a reminder of the conditioned association from day 1. tDCS was applied during extinction. On the third day, stimuli were again presented without the US to assess extinction recall. SCR and self-reported fear served as indicators of conditioned fear. No significant effects of the intervention were detected during the extinction phase. During the recall phase, the results showed significant changes in SCR and self-report measures. The SCR response to CS+ in the anodal tDCS group was comparable to the CS- response, and furthermore, self-reported fear showed retention of fear related to the CS+, as compared to sham. Thus, tDCS led to overgeneralization of the vegetative fear response to non-reinforced stimuli. van't Wout et al. (2016) conducted a two-day protocol study to assess the impact of tDCS on fear extinction and recall. The anodal electrode was placed over the left vmPFC (return electrode over contralateral mastoid), and stimulation was conducted with 2 mA for 10 min. Participants underwent habituation, acquisition, and extinction phases during the first day. Each participant was conditioned to two CS+, and each CS+ was presented in one of two consecutive extinction blocks. The CS were presented in different contexts according to phase of the protocol (i.e., one context during acquisition and another during extinction and recall). An electrical shock served as US, the conditioned stimuli were colored lights. The first group received 5 min of tDCS before extinction, and stimulation continued the next 5 min during the first extinction block. In the second block, sham stimulation started 5 min before the second extinction block and continued during extinction. The second group received the reversed order of stimulation. In this design, each participant received anodal tDCS during the extinction of one CS+ and sham stimulation during the extinction of the other CS+. During the second day, extinction recall was assessed. SCR was used as a dependent measure. Participants who received tDCS during the first extinction block showed lower SCR during late extinction of the second extinction block. No effect of tDCS on extinction recall was observed. Another study that assessed the effect of tDCS on fear extinction was conducted by Dittert et al. (2018). Participants received bilateral stimulation (i.e., right anodal- left cathodal tDCS, and vice versa) or respective sham stimulation over the vmPFC in a one-day protocol (i.e., habituation, acquisition, and extinction performed on the same day). Duration of stimulation was 20 min, and the intensity of the applied stimulation was 1.5 mA. Two neutral female faces served as CS, and a female scream simultaneously presented with a fearful expression of the face was used as the US. Two active stimulation groups were treated with the same electrode positions, but opposite current flow directions. The stimulation started during a 10 min break between acquisition and extinction and went on until the end of extinction. SCR, self-report measures [subjective ratings of arousal, valence, and CS-US contingency, STAI-X1 (Laux et al., 1981) and PANAS (Watson et al., 1988)] served as dependent variables. tDCS accelerated early extinction learning in both real stimulation groups. Furthermore, the significant decrease of reaction toward the CS+ was accompanied by an increased reaction toward the CS– in the active tDCS groups. Furthermore, the left anodal tDCS group showed a higher decrease in subjectively rated state anxiety. Vicario et al. (2020b) conducted a sham-controlled study with a 2-day fear extinction protocol to investigate the effect of anodal tDCS on fear extinction. Anodal tDCS was applied over the left vmPFC (return electrode over controlateral mastoid) with an intensity of 2 mA for 10 min. During the first day participants underwent habituation, acquisition, and extinction stages. Two colored circles were used as a CS and one of them was followed by a highly uncomfortable electrical stimulus. Stimulation was applied during the whole extinction phase. Fear responses were assessed by SCR. Anodal tDCS over the left vmPFC reduced fear reactions during extinction recall in participants that acquired fear responses during fear acquisition. Results of electrical field simulations showed that the AF3-contralateral mastoid montage used in this study is better suited than other protocols to tackle the vmPFC, and amygdala, which are both crucial for extinction learning. Ney et al. (2021) conducted a sham-controlled study with a 2-day fear extinction protocol to investigate how timing of tDCS will influence fear extinction retention. The fear conditioning/extinction protocol and stimulation parameters were identical to those in the previous study (Vicario et al., 2020b), but tDCS or sham stimulation were applied 10 min after fear extinction to target consolidation processes. Fear responses were assessed by SCR. In that study, anodal stimulation led to impaired fear extinction retention.

One study has targeted the dlPFC to modulate fear extinction. Ganho-Ávila et al. (2019) conducted a study in female participants using a 3-day paradigm to investigate the effects of cathodal tDCS on fear extinction. Cathodal stimulation was delivered over the right dlPFC (return electrode over contralateral deltoid) for 20 min at 1 mA intensity. On the first day, habituation and fear acquisition were conducted. The authors used two colors as CS and a female scream as the US. On the second day, before tDCS, participants were asked to verbally recall the CS+, and afterwards stimulation was applied. Then extinction learning was conducted. After 1 to 3 months, participants participated in follow-up sessions. Participants were asked to recall the CS+, and were again exposed to four unsignaled USs. The re-extinction phase started immediately after reinstatement. To assess fear conditioning/extinction learning, SCR, and self-reports (valence, arousal, contingency, and expectancy) were conducted. Furthermore, the State-trait anxiety inventory (STAI-S; Spielberger, 1984) and Approach avoidance task (AAT; Krypotos et al., 2014), which is designed to assess implicit avoidance tendencies, were employed. Cathodal tDCS had no immediate effect on SCR and self-report measures. The delayed after-measures showed however increased CS+ retention, suggesting a reduction of extinction efficacy by cathodal tDCS. Moreover, cathodal tDCS enhanced CS+/CS– stimuli discrimination, as measured by the AAT task, via establishing a positive bias toward the CS–, leading to a decreased generalization effect.

Taken together, the results of these studies suggest that tDCS over the vmPFC can influence fear extinction and recall in healthy humans. Specifically, studies suggest that anodal tDCS over the vmPFC leads to enhanced fear extinction memory consolidation, but these effects seem to critically depend on experimental protocol characteristics, as well as timing and area of stimulation. Diminution of activity of the dlPFC seems to reduce extinction and enhance CS+/CS– discrimination.

#### Effects of tDCS on Fear Extinction in the Clinical Population

One study was identified that meets the described criteria (Table 3). van't Wout et al. (2017) conducted a 2-day fear extinction protocol in male veterans with posttraumatic stress disorder (PTSD) to assess effects of tDCS, and timing of stimulation (i.e., during or after extinction) on fear extinction memory. Anodal tDCS was conducted over the left vmPFC (return electrode over controlateral mastoid) for 10 min with an intensity of 2 mA. On the first day, participants underwent habituation, acquisition and extinction. Different contexts were used for acquisition on the one hand, and extinction and recall on the other, and each participant was conditioned to two different CS. An electrical shock was used as US. Half of the participants were stimulated with tDCS during extinction and half of them immediately after extinction. On the second day, extinction recall was performed. SCR served as dependent measure. Veterans who received anodal tDCS after fear extinction showed trendwise lower SCR on early recall, compared to those who received stimulation during extinction learning.

### NIBS and Exposure Therapy

#### rTMS and Exposure Therapy

Nine articles were identified which met the inclusion criteria (Table 5). Two studies adopted rTMS and exposure protocols in patients with specific phobias, three in patients with PTSD and four in patients with OCD. Notzon et al. (2015) conducted a sham-controlled study on patients with spider phobia to assess the combined effect of intermittent Theta Burst Stimulation (iTBS) and exposure on symptoms. Participants received facilitatory iTBS or sham stimulation over the left dlPFC before VR spider exposure. For assessment of spider fear symptoms, the Fear of Spiders Questionnaire (FSQ; Szymanski and O'Donohue, 1995), and Spider Phobia Questionnaire (SPQ; Olatunji et al., 2009) were used. Besides that, the Anxiety Sensitivity Index (ASI; Reiss et al., 1986), the questionnaire for the assessment of disgust sensitivity (disgust scale: DS; Haidt et al., 1994), the Subjective Units of Discomfort Scale (SUDS; Wolpe, 1973), and also electrophysiological measures of HR, heart rate variability (HRV) and SCR were conducted. The results showed no effect of iTBS on self-report measures of anxiety and disgust of spiders, HR, and SCR. Regarding HRV, iTBS significantly increased sympathetic activity during the spider scene. Herrmann et al. (2017) conducted a sham-controlled study to investigate the effects of rTMS on height phobia. Participants were exposed to two virtual reality scenarios within a period of 2 weeks, and before each VR session, facilitatory rTMS (10 Hz, 100% RMT), or sham stimulation was applied bilaterally over the mPFC for 20 min. The Acrophobia Questionnaire (AQ; Cohen, 1977), and Behavioral Avoidance Test served as outcome measures. The results show a significant reduction of phobic anxiety and avoidance measured by the AQ in active-, as compared to sham-stimulated patients immediately but not 3 months after intervention.

Osuch et al. (2009) performed a study to assess the potential of rTMS to reduce symptoms in patients with chronic PTSD. In a sham-controlled crossover design, patients received one block of 20 sham rTMS and exposure sessions and one block of 20 active rTMS and exposure sessions. Low-frequency inhibitory rTMS (1 Hz, 100% RMT) was delivered over the right dlPFC. Before the treatment, each participant completed a list of 10 events or cues that were used during the exposure session (i.e., experience 0–referred to something calming, experience 1–referred to a neutral experience, experiences 2–9 were related to the trauma). During the first and second sessions in each condition, subjects were instructed to talk about item number 0 and item number 1, respectively, for 5 min in order to become habituated to the experimental setting. In subsequent sessions, subjects could freely choose to speak 5 min about any of the 10 items on their list or remain silent, but they could talk more if they wanted. Stimulation was delivered 5 min before participants started to speak about the events and lasted for 30 min. As outcome measures, the Clinician Administered PTSD Scale (CAPS; Blake et al., 1995), the Impact of Event Scale (IES; Sundin and Horowitz, 2002), and the Hamilton Depression Rating Scale (HDRS; Hamilton, 1960) were applied at baseline and after treatment. Combination of active rTMS and exposure led to a moderate improvement of hyperarousal symptoms assessed by the CAPS. A study by Isserles et al. (2013) assessed the effect of deep rTMS on symptom improvement in pharmaco- and psychotherapy-resistant PTSD patients. Facilitatory stimulation was delivered over the mPFC at 20 Hz frequency (120% RMT). One group received deep rTMS after script-driven imagery of a traumatic experience, the second group received deep rTMS after script-driven imagery of a positive experience, and the third group received sham rTMS after script-driven imagery of traumatic experiences. The participants received 3 treatment sessions per week during a period of 4 weeks. Each session lasted for around 20 min with ~4 min of script-driven imagery followed by 15.5 min of deep rTMS. The authors performed the Clinically Administered PTSD Scale (CAPS; Blake et al., 1995) at baseline, and at the 5th, 7th and 13th week as primary outcome measure for assessing PTSD symptoms. Additionally, the PTSD-symptoms scale-self report (PSS-SR; Foa et al., 1993), HDRS-24 (Hamilton, 1960), and the Beck Depression Inventory-II (BDI-II; Beck et al., 1996) were conducted at baseline, once weekly at the beginning of each treatment week, at the end of the treatment phase at week 5, and at 7th and 13th week for follow up. Furthermore, HR was recorded before, during and after each script imagery period. The traumatic experience imagery group exposed to active rTMS significantly improved in total CAPS and corresponding domain scores (i.e., intrusion, avoidance/numbness, and arousal), compared to the other two groups. This beneficial effect was preserved during the follow-up period. Even for the secondary outcome measures (i.e., PSS-SR, HDRS-24, and BDI-II), symptom improvements were obtained in the traumatic experience imagery group exposed to active rTMS during treatment and follow-up periods. Furthermore, HR was significantly reduced throughout treatment in the traumatic experience imagery group exposed to active rTMS. Fryml et al. (2019) performed a study in which facilitatory rTMS (10 Hz, 120% RMT) was performed over the left or right dlPFC. Participants were furthermore divided into active rTMS and sham groups and treated one time per week (for 5 weeks), combined with imaginal exposure to traumatic situations. The whole session lasted around 40 min with rTMS started 5 min after the begin of exposure for a total duration of 30 min. The Clinically Administered PTSD Scale (CAPS; Blake et al., 1995) and Hamilton Rating Scale for Depression (HRSD; Hamilton, 1960) were applied as outcome measures. CAPS scores showed a trend toward improvement in the real vs. sham rTMS condition after the treatment. Interestingly, the active rTMS group had furthermore significantly lower depression scores at the fourth and fifth sessions relative to baseline and compared to sham. The authors did not report whether any differences were found regarding the area of the stimulation (i.e., right vs. left dlPFC).

Carmi et al. (2018) performed a study on OCD patients to assess whether high or low frequency deep rTMS affects symptoms. Three groups of patients received high frequency rTMS (20 Hz, 100% RMT), low frequency rTMS (1 Hz, 110% RMT), or sham rTMS applied bilaterally to tackle the mPFC and ACC. Each treatment session began with a 3–5 min provocation of personalized obsessive-compulsive cues and stimulation was delivered afterwards. Patients were treated five times per week for 5 weeks (25 sessions in total). As outcome measures, the Yale-Brown Obsessive-Compulsive Scale (YBOCS; Goodman et al., 1989), and Clinical Global Impression Scale-Improvement (CGI-I; Guy, 1976) were performed at baseline (pre-treatment), during and up to 1 month after intervention. YBOCS and CGI-I scores improved by high-frequency deep rTMS in contrast to low frequency and sham interventions, and the effect was significant for 1 week, but not for 1 month after intervention. In another sham-controlled study, Carmi et al. (2019) assessed the effect of high frequency deep rTMS for the treatment of OCD patients. Parameters and area of rTMS, and the exposure protocol were identical to those described in the previous study, except that the treatment lasted for 6 weeks, and included a 4-week follow-up. The Yale-Brown Obsessive-Compulsive Scale (YBOCS; Goodman et al., 1989), Clinical Global Impression Scale-Improvement (CGI-I; Guy, 1976), Clinical Global Impressions Severity scale (CGI-S; Guy, 1976), and Sheehan Disability Scale (Sheehan et al., 1996) were applied for obtaining outcome measures. The results showed a significant reduction of OCD symptoms in the active as compared to the sham rTMS group at the posttreatment assessment and the 4-week follow-up. Furthermore, global functioning was improved by active rTMS, as compared to sham treatment at the posttreatment assessment. Furthermore, two case studies combined rTMS and exposure protocols to reduce OCD symptoms. Adams et al. (2014) combined exposure and response prevention (ERP, see Foa et al., 2005) with low frequency rTMS (1 Hz pulses, 110% RMT) over the pre-supplementary motor area to treat a male patient that showed minimal response to medication. Low-frequency rTMS was delivered immediately prior to ERP for 3 weeks. The results showed improvement in OCD (YBOCS; Goodman et al., 1989, DOCS; Abramowitz et al., 2010), generalized anxiety (GAD-7; Spitzer et al., 2006) and depression symptoms (PHQ-9; Kroenke et al., 2001). Grassi et al. (2015) investigated the effects of high-frequency rTMS (10 Hz frequency, 80% of RMT) applied over the left dlPFC for 10 session of a treatment-resistant OCD patient. Each exposure session was immediately preceded by stimulation. Authors reported a reduction of symptom severity measured by the Y-BOCS (Goodman et al., 1989), CGI-I (Guy, 1976) and Global Assessment of Functioning (GAF) scales. The clinical improvement was maintained, and the global level of functioning increased for up to 24 months after intervention.

In general, the results of the above-mentioned studies suggest a potential of rTMS in combination with exposure protocols to reduce symptoms in individuals with specific phobias, PTSD and OCD. In patients with specific phobias, results suggest that high frequency rTMS over the mPFC might be promising. For PTSD treatment, stimulation over the dlPFC and mPFC have shown effects. High frequency deep rTMS over the mPFC and ACC shows promising results for treatment of OCD patients. Here, pre-supplementary motor area and left dlPFC stimulation might also be promising.

#### tDCS and Exposure Therapy

Two studies explored the effect of exposure therapy combined with tDCS (Table 3). van't Wout-Frank et al. (2019) assessed anxiolytic effects of tDCS combined with Virtual reality (VR) exposure in veterans with warzone-related PTSD. Anodal tDCS was applied over the left vmPFC (return electrode over the contralateral mastoid) for 25 min at an intensity of 2 mA. Participants received active or sham tDCS in 6 sessions during exposure to three VR driving scenarios (8 min duration), in which 12 warzone events were presented. A head-mounted display with integrated head tracking and stereo earphones presented combat-related multisensory information (visual, auditory, olfactory, and haptic). Measures of SCR and self-reported PTSD symptoms (at baseline, after VR sessions, and 1 month later) were obtained. SCR was reduced to a larger extent in the active tDCS group, as compared to sham. Both groups showed furthermore a significant reduction in PTSD symptom severity after treatment, but only the active tDCS group continued to improve during the 1-month follow-up. Todder et al. (2018) performed a sham-controlled crossover study in refractory OCD patients i to assess whether anodal or cathodal tDCS applied bilaterally over the mPFC (return electrode over right shoulder) for 20 min per session at an intensity of 2 mA reduces obsession-induced anxiety. During the 5 weeks of treatment (first, third and fifth week were treatment sessions), participants received anodal, cathodal, or sham tDCS three times a week with 48-h intervals in-between. Before the first session, obsession-provoking stimuli (OPS) were individualized for each patient. In all following sessions, patients were first exposed to OPS, and then to tDCS for 20 min. Participants rated their level of anxiety via a Visual Analog Scale (VAS) immediately after OPS and tDCS. Additionally, the clinical scales CGI (Guy, 1976), Montgomery-Åsberg Depression Rating Scale (MADRS; Montgomery and Åsberg, 1977), HAM-A (Hamilton, 1960), Y-BOCS (Goodman et al., 1989) were rated by the patients at the beginning of each stimulation week. Cathodal tDCS reduced obsession-induced anxiety, as compared to anodal and sham stimulation.

In summary, these results suggest that the combination of tDCS over the mPFC and exposure has potential to reduce PTSD, and OCD symptoms. The effect of tDCS over other areas on respective symptoms has however not been explored, and the efficient stimulation protocols differed between diseases, and timing of stimulation.

## Discussion

We systematically reviewed 30 research articles conducted in animal models and healthy humans, but also clinical patient groups that aimed to influence fear and extinction memory processes via NIBS methods. In summary, the reviewed articles show a potential of NIBS to influence fear memory, enhance fear extinction and reduce clinical symptoms in various fear-related disorders. The potential and limitations of these studies will be discussed in the next paragraphs.

### Fear Memory

The prefrontal cortex plays an important role in controlling several cognitive and affective functions (e.g., Ridderinkhof et al., 2004). In this regard, the dlPFC is assumed to be critically involved in up-/down regulation of the cortico-meso-limbic network (Vicario et al., 2019), and shows activity enhancement during fear conditioning (Dunsmoor et al., 2007, 2008). In contrast, the vmPFC is relevant for the up-regulation of reward seeking behavior (Hutcherson et al., 2012), down-regulation of negative affective responses (Diekhof et al., 2011), and critically involved in extinction learning and recall (Phelps et al., 2004; Milad et al., 2007). Accordingly, in most of the studies the dlPFC was selected as target area for modulation of fear memory.

In general, the examined literature on fear memory is limited in terms of studies which tackled specific areas. Regarding animal studies, it offers only investigations with tDCS over the PFC, with limited specification of different subregions of this cortical target. Overall, the main results suggest that tDCS can alter fear memories, but specific effects are intervention timing- and brain state-related. PFC stimulation (both anodal and cathodal) immediately before fear acquisition reduced fear memory (i.e., Abbasi et al., 2017). In other studies, stimulation effects were reported also when tDCS was performed after conditioning. Moreover, for pharmacologically impaired fear learning, tDCS restituted learning, when stimulation was performed 24 h before conditioning, which shows a dependency of the directionality of effects on brain states.

In humans, excitability-diminishing cathodal tDCS over the left dlPFC disrupted fear memory consolidation, while excitability-enhancing anodal tDCS had no effects (Asthana et al., 2013). This result is in accordance with an rTMS study showing that inhibitory rTMS over the left dlPFC disrupts fear memory consolidation (Borgomaneri et al., 2020). In contrast, excitability-enhancing anodal, but not cathodal tDCS over the right dlPFC enhanced fear memory (Mungee et al., 2014, 2016) when applied during the reconsolidation period. However, in the latter study the return electrode was placed over the vmPFC, another cortical region relevant for memory consolidation (Nieuwenhuis and Takashima, 2011), which makes interpretation of the results of that study complex.

Mechanistically, the impact of left dlPFC modulation on fear memory can be explained by referral to the neuroimaging literature on memory consolidation. It has been suggested that the dlPFC is functionally connected with the hippocampus (Wang and Morris, 2010). Liu et al. (2016) reported that an attenuated hippocampal functional connectivity with the left dlPFC was predictive of more effective suppression of overnight consolidation of aversive memories. Accordingly, inhibitory tDCS, and rTMS over the left dlPFC might have resulted in attenuated hippocampal activity, and thus impaired consolidation. On the other hand, the enhanced fear memory following excitatory right dlPFC stimulation (Mungee et al., 2014) is in line with evidence for higher activation of the right dlPFC during memory retrieval processes (Sakai et al., 1998). Overall, these studies suggest that the dlPFC can be a relevant cortical target for fear memory consolidation.

A limitation of the NIBS literature on fear memory is the absence of “online” stimulation protocols, that should have also tackled the fear acquisition stage, besides consolidation, and re-consolidation. Future research should close this gap, although consolidation studies might be particularly promising from a clinical point of view (e.g., early intervention after trauma). Moreover, studies are needed to more systematically explore functional differences between the left and the right hemisphere, to clarify the optimal timing of stimulation with respect to the considered process (i.e., acquisition, consolidation, reconsolidation) and clarify the underlying specific mechanism. Finally, comparability between studies conducted in animal models and humans is currently limited because of relevant differences between the respective protocols (including targeted cortical sites, and the use of pharmacological manipulations in animal models).

### Fear Extinction

Studies on human and animal models have demonstrated that rTMS and tDCS can lead to enhancement of fear extinction and affect related fear circuits. Previous imaging studies have shown that the vmPFC is specifically activated during fear extinction (Phelps et al., 2004; Milad et al., 2007), and this area is assumed to be involved in the top-down regulation of the amygdala during extinction learning (Milad et al., 2007). Therefore, inducing LTP-like plasticity over the vmPFC by NIBS methods is assumed to enhance regulation of the amygdala and lead to reduction of fear expression.

#### tDCS

##### Area of Stimulation

In most studies (five out of six that have employed a fear extinction protocol), application of tDCS in healthy humans over the vmPFC and mPFC resulted in enhanced fear extinction learning and memory. In accordance, anodal tDCS over the vmPFC improved fear extinction and recall in healthy humans (van't Wout et al., 2016; Dittert et al., 2018; Vicario et al., 2020b), and PTSD patients (van't Wout et al., 2017). In contrast to these results, Abend et al. (2016) reported detrimental effects of anodal tDCS over the mPFC, i.e., an overgeneralization of fear response by tDCS. Dittert et al. (2018) also reported a gradual enhancement of the vegetative reaction to the non-reinforced stimuli in addition to the extinction enhancement induced by tDCS, which stresses the importance of further research for optimizing stimulation parameters. The specific electrode arrangement used in the Abend et al. (2016) study might explain its deviating results. Computational modeling results (Vicario et al., 2020b) suggest that the AF3/Mastoid electrode montage, which was applied in the studies conducted by van't Wout et al. (2016), and Vicario et al. (2020b) result in stronger electrical fields at the level of the vmPFC and amygdala, as compared to the FPz/Iz montage used by Abend et al. (2016).

Only one study is available that applied cathodal tDCS over the right dlPFC for extinction modulation (Ganho-Ávila et al., 2019). The results of this study show a delayed enhancement of fear memory and CS+/CS– discrimination, which suggest a positive effect of this stimulation protocol with respect to specification of a stimulus as dangerous, or not. This enhanced discrimination by cathodal tDCS might be related to attentional processes that improved signal to noise discrimination, as shown already for visuo-motor learning.

In line with anatomical, optogenetic, lesion and imaging studies (Quirk et al., 2000; Phelps et al., 2004; Milad et al., 2007; Motzkin et al., 2015; Kim et al., 2016), available tDCS studies favor the vmPFC as target for stimulation in order to enhance fear extinction and recall. Studies on other areas are largely missing due to conceptual reasons, or because these areas are not surface-near, and thus no suitable target for NIBS approaches. The at least partial heterogeneity of the results of different studies is likely caused by protocol differences, and we will focus on relevant parameters in the next sections.

##### Timing of Stimulation

An important factor that affects the results of stimulation is whether tDCS is delivered during extinction (i.e., online) or before or after extinction (i.e., offline), because the timing of stimulation determines how respective stimulation- and task-dependent physiological effects interact. The three studies conducted in humans (i.e., three anodal tDCS protocols–van't Wout et al., 2016; Dittert et al., 2018; Vicario et al., 2020b), that have applied online stimulation over the vmPFC or areas closely connected with the vmPFC improved extinction. Ney et al. (2021) showed detrimental effects of anodal tDCS over the vmPFC on fear extinction retention, when the consolidation window was targeted, which further emphasizes the importance of appropriate timing of stimulation. This general pattern of results is supported by optogenetic studies (Do-Monte et al., 2015). In general accordance, online tDCS, compared with offline tDCS, has been shown to have a superior impact on various tasks (Stagg et al., 2011; Martin et al., 2014; Dedoncker et al., 2016; Oldrati et al., 2018). Therefore, enhancing LTP-like plasticity of fear-related brain circuits during extinction learning might be advantageous. In contrast (van't Wout et al., 2017), one study in PTSD patients, that tested whether anodal tDCS over the vmPFC has better effects when applied online or offline, showed enhanced extinction recall when tDCS was applied after extinction, as compared to online stimulation. The lack of a control group, small sample size and recruitment of exclusively male participants in this study limits conclusions, but it might be that stimulation after extinction leads to enhanced consolidation of fear extinction memory, which should be tested in future studies. Given the partially heterogeneous study results, a systematic evaluation of the optimal timing of intervention is still warranted. Specifically, research protocols are needed to clarify the different role of encoding and consolidation of fear extinction memory (Vicario et al., 2017) and directly test, in otherwise identical protocols, the effect of online vs. offline stimulation. Here, an important point regarding consolidation of fear and extinction memory is the time interval between acquisition and extinction (Abend and van't Wout, 2018). If not sufficiently temporally discerned, stimulation might modulate fear related memory that is not yet consolidated, which could lead to mixed effects. For example, traumatic events are usually separated, and therefore consolidated, prior to pharmacological or psychotherapeutic interventions. Therefore, using protocols where acquisition of fear is not immediately followed by extinction might lead to more ecologically valid results.

##### Duration and Intensity of Stimulation

Especially for tDCS, duration and intensity of interventions varied relevantly between studies. Positive effects on fear extinction and recall were observed with anodal tDCS at an intensity of 2 mA (van't Wout et al., 2016, 2017; Vicario et al., 2020b) applied for 10 min, but also 1.5 mA (Dittert et al., 2018) applied for 20 min, and with different electrode sizes. Since duration and intensity of stimulation influence cortical excitability in a partially non-linear manner (Batsikadze et al., 2013; Jamil et al., 2017; Agboada et al., 2019; Samani et al., 2019), further research should address the relationship between these factors and fear extinction. Furthermore, none of the studies have applied anodal stimulation with an intensity of 3 mA over the vmPFC, that according to recent studies might be more efficient than lower stimulation intensities (Agboada et al., 2019). Since the vmPFC is not a surface-near structure, increasing stimulation intensity might lead to better activation of this area that could in turn lead to enhanced extinction and retrieval of fear extinction memory. Furthermore, the tDCS studies varied with respect to the applied current densities, which could have an impact on the results. Future studies should test systematically how different intensities and durations of applied current relate to fear extinction.

##### Hemispheric Lateralization

Only one study has directly compared the effect of left anodal—right cathodal, and vice versa tDCS stimulation protocols and provided some insights into prefrontal hemispheric lateralization (Dittert et al., 2018). No current flow direction-dependent effects on early extinction were detected but left anodal tDCS reduced additionally state anxiety in that study. Furthermore, three more studies (van't Wout et al., 2016, 2017; Vicario et al., 2020b) that have applied anodal tDCS over the left vmPFC (anode over the AF3 and cathode over the contralateral mastoid process). A hemispheric lateralization of the PFC in emotion regulation has been previously documented, relating activity of the left PFC to the ability to adequately regulate emotions (Kim and Bell, 2006). In accordance, it has been demonstrated that the metabolic activity of the left PFC is increased in persons that use reappraisal strategies, which is positively related to greater experience and behavioral expression of positive emotion and increased sense of well-being, while suppression of emotional expressions was negatively associated with reduced right-hemispheric glucose metabolism (Kim et al., 2012). Furthermore, emotion regulation is accompanied by enhanced left hemispheric connectivity between the amygdala, and the vmPFC, OFC, dmPFC, and dlPFC in persons with high reappraisal use (Eden et al., 2015). Therefore, it might be assumed that anodal tDCS over the left vmPFC leads to enhanced emotion regulation. In contrast, Dittert et al. (2018) found that right anodal-left cathodal stimulation might be advantageous in reducing fear responses in late extinction blocks. Considering these preliminary and partially conflicting results, further research is required to explore if hemispheric lateralization is an important factor in regulating extinction.

#### rTMS

##### Area of Stimulation

The rTMS studies document enhanced fear extinction learning and memory following stimulation of the vmPFC and mPFC in healthy humans. Specifically, high-frequency, excitability-enhancing rTMS (Klomjai et al., 2015) over the mPFC and vmPFC reduced fear responses (Guhn et al., 2014; Raij et al., 2018). This pattern of results is supported by studies in animal models which showed that apart from enhancing fear extinction (Baek et al., 2012; Legrand et al., 2019), application of high frequency rTMS over the PFC induced structural changes, and alterations of early gene expression in the infralimbic cortex (functionally related to the human vmPFC), the basolateral amygdala and the ventral CA1, which are relevant for extinction of fear memories (Legrand et al., 2019). Overall, the results from the examined rTMS study corroborates those of respective tDCS studies.

##### Timing of Stimulation

One excitatory rTMS study–(Raij et al., 2018) with online stimulation over the vmPFC (however with an indirect approach with direct stimulation of a lateral PFC area closely connected with the vmPFC) report improved extinction. This result is supported by a rTMS study in an animal model, where online stimulation of the vmPFC had better outcomes as compared to offline stimulation (Baek et al., 2012). On the other hand, Guhn et al. (2014) provide evidence of effective fear extinction following excitatory stimulation over the mPFC, and thus with offline stimulation.

Overall, the results from the available rTMS studies suggest that both online and offline stimulation can be effective in boosting fear extinction, depending on the considered cortical target. More research is needed to explore the effectiveness of online and offline interventions regarding different cortical targets.

##### Duration and Intensity of Stimulation

Two rTMS studies have applied relevantly different facilitatory stimulation protocols with respect to intensity and duration, but also timing (Guhn et al., 2014; Raij et al., 2018). Since duration and intensity of stimulation influence cortical excitability (Fitzgerald et al., 2006; Lang et al., 2006), further research should address the relationship between these factors and fear extinction systematically.

##### Hemispheric Lateralization

Only one study (Raij et al., 2018) suggests an impact of hemispheric lateralization on rTMS results. In that study, application of rTMS over the left posterior PFC reduced fear reactions. This finding is in line with tDCS studies on fear extinction and recall. Lateralized effects thus might be assumed, but were not systematically studied with rTMS. Overall, these results are preliminary, and more work is needed to explore the relevance of hemispheric lateralization with respect to fear extinction.

#### Methodological Considerations

Fear memory and extinction studies vary relevantly with respect to fear conditioning-extinction protocol characteristics, which complicate interpretation of outcomes. Reinforcement rates in studies discussed here vary from 38 to 80%. It has been previously demonstrated that different reinforcement rates can relevantly influence fear responses (Chin et al., 2016), and therefore, this might be a factor that influences the results of interventions. Also, the modality of US stimuli (i.e., scream vs. electrical shock) that was used for fear conditioning might affect results. Larger startle responses are exhibited in the electrical shock task, and the respective US shock and overall task are rated as more aversive than the scream (Glenn et al., 2012). Furthermore, some studies used CS+ reminders before extinction (Abend et al., 2016; Ganho-Ávila et al., 2019), which could affect the outcomes of extinction learning by re-activation of fear memories. Intervals between acquisition and extinction varied between studies, which could further influence results via an effect of stimulation on respective re-activated memory traces. Considering that stimuli and procedures used in fear extinction protocols cannot encompass all aspects and the complexity of fear emotion and anxiety in the real world, moving toward more ecologically valid protocols might lead to more relevant outcomes. Virtual reality might be a promising tool as it combines the experimental control of laboratory measures with real life scenarios providing immersion, presence, impact on different sensory modalities, control of actions and interactions (Parsons, 2015; Carl et al., 2019).

### NIBS and Exposure Protocols

Studies exploring the effect of rTMS and tDCS combined with exposure protocols show a potential to improve symptoms in specific phobias, PTSD and OCD. Similar to what was discussed in the previous section, however also here heterogeneities of protocols make it difficult to come to definite conclusions.

For specific phobias, one study that applied facilitatory rTMS over the vmPFC combined with exposition reduced symptoms in patients with height phobia (Herrmann et al., 2017). Notzon et al. (2015), on the contrary, did not find a significant symptom improvement in patients with spider phobia by excitability-enhancing iTBS over the dlPFC. Reasons for these discrepant results could be that Herrmann et al. (2017) applied repeated stimulation over the vmPFC, while Notzon et al. (2015) conducted a single session approach over the left dlPFC, and that the vmPFC, but not the dlPFC is assumed to have a critical role in extinction. Moreover, the specific stimulation protocol differed between studies.

A couple of studies has been conducted in PTSD patients. Facilitatory (Isserles et al., 2013) rTMS over the mPFC and inhibitory (Osuch et al., 2009) rTMS applied over the dlPFC combined with exposure have shown to improve symptoms. Fryml et al. (2019) did not find an effect of facilitatory rTMS over the dlPFC on PTSD symptoms. Beyond rTMS, also anodal tDCS over the left vmPFC combined with VR exposition reduced SCR and symptoms in veterans with warzone-related PTSD (van't Wout-Frank et al., 2019). This study supports findings that online and left vmPFC application of tDCS is a promising way for enhancing fear extinction. Overall, these studies show that enhancing LTP-like plasticity over the vmPFC with rTMS and tDCS, leads to enhanced fear extinction and symptom improvement. Furthermore, inhibitory stimulation over the dlPFC might improve symptoms. Due to the preliminary and partially mixed results of the available data, and missing comparative studies, firm conclusions about the efficacy of specific stimulation parameters are difficult to make, and future studies should explore these aspects systematically.

For OCD treatment, preliminary results suggest furthermore that high frequency deep rTMS targeting the mPFC and ACC reduces symptoms in these patients (Carmi et al., 2018, 2019). Additionally, in two case studies, symptoms improved after low frequency rTMS over the pre-supplementary motor area (SMA) and high frequency rTMS over the left dlPFC (Adams et al., 2014; Grassi et al., 2015). Interestingly, in difference to the respective rTMS-studies, where excitability-enhancing stimulation over the mPFC reduced symptoms, cathodal, but not anodal tDCS over the mPFC reduced obsession-induced anxiety in OCD patients (Todder et al., 2018). One explanation for these seemingly conflicting results might be application of different NIBS methods (i.e., deep rTMS might lead to deeper penetration of the brain) and different areas of stimulation. Furthermore, case studies suggest that excitatory stimulation over the left dlPFC might lead to enhanced cognitive control in OCD, while inhibition of the pre-SMA, whose hyperactivity underlies cognitive control deficits in OCD (Adams et al., 2014), lead to symptom reduction. Therefore, inducing LTP- or LTD-like plasticity with tDCS and rTMS over the PFC might be a promising way to alleviate symptoms in OCD, however available data are scarce. Future studies should further explore and optimize parameters of stimulation (e.g., area, frequency, duration, excitation, or inhibition inducing methods) to develop more efficient treatments.

For mechanisms of these effects, exposure protocols might lead to activation of symptom-related neural circuits (Carmi et al., 2018, 2019) engaged in dysfunctional cognition, which are then susceptible to change via application of NIBS. In line with previous claims that online stimulation might have advantages as compared to offline interventions, combining exposure protocols with simultaneous NIBS might have a better outcome than application of stimulation alone, or conducted before exposure. On the other side, previous studies have shown that application of rTMS or tDCS also without exposure can lead to symptom reduction (Vicario et al., 2019) and, therefore, future studies should compare effects of these two approaches. Furthermore, disease-related hyper- or hypo-activation of specific brain areas might depend critically on the respective disease, and therefore designing stimulation parameters optimized for specific disorders, or symptoms, is important. For example, based on the results mentioned above, enhancing excitability in medial parts of the PFC, and reducing it in pre-SMA might lead to better outcomes in patients with OCD.

## General Remarks

**Figure 2:**
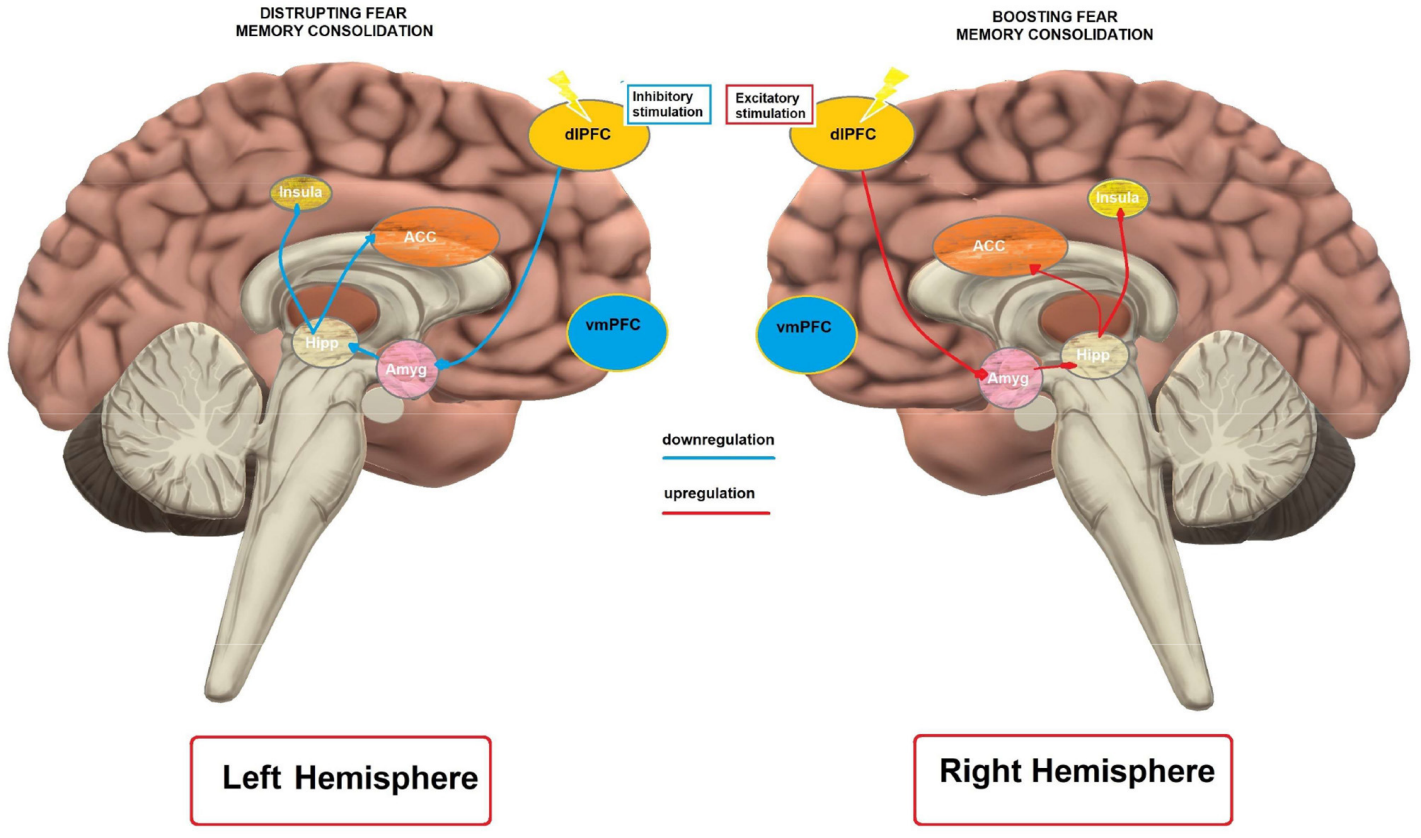
Model on how NIBS is thought to influence fear memory formation. Inhibitory NIBS of left dlPFC interferes with fear memory consolidation by downregulating the activity of the amygdala which, in turn, downregulates the hippocampus, which is responsible for memory consolidation, and ACC/insula, which are involved in the expression of fear responses. Excitatory NIBS of the right dlPFC boosts fear memory consolidation by upregulating the activity of amygdala, which, in turn, upregulates the activity of hippocampus and ACC/Insula.

The present review provides evidence that NIBS methods are promising to influence fear memory and fear extinction processes and have potential for the treatment of various clinical fear-related syndromes. Our work provides preliminary evidence linking the dlPFC with fear memory in humans (Figure 2), probably at the level of consolidation/reconsolidation processes. However, results from research with animal models provide a mixed picture, which might be due to substantial protocol differences (including differences of cortical stimulation sites and use of pharmacological manipulation).

**Figure 3:**
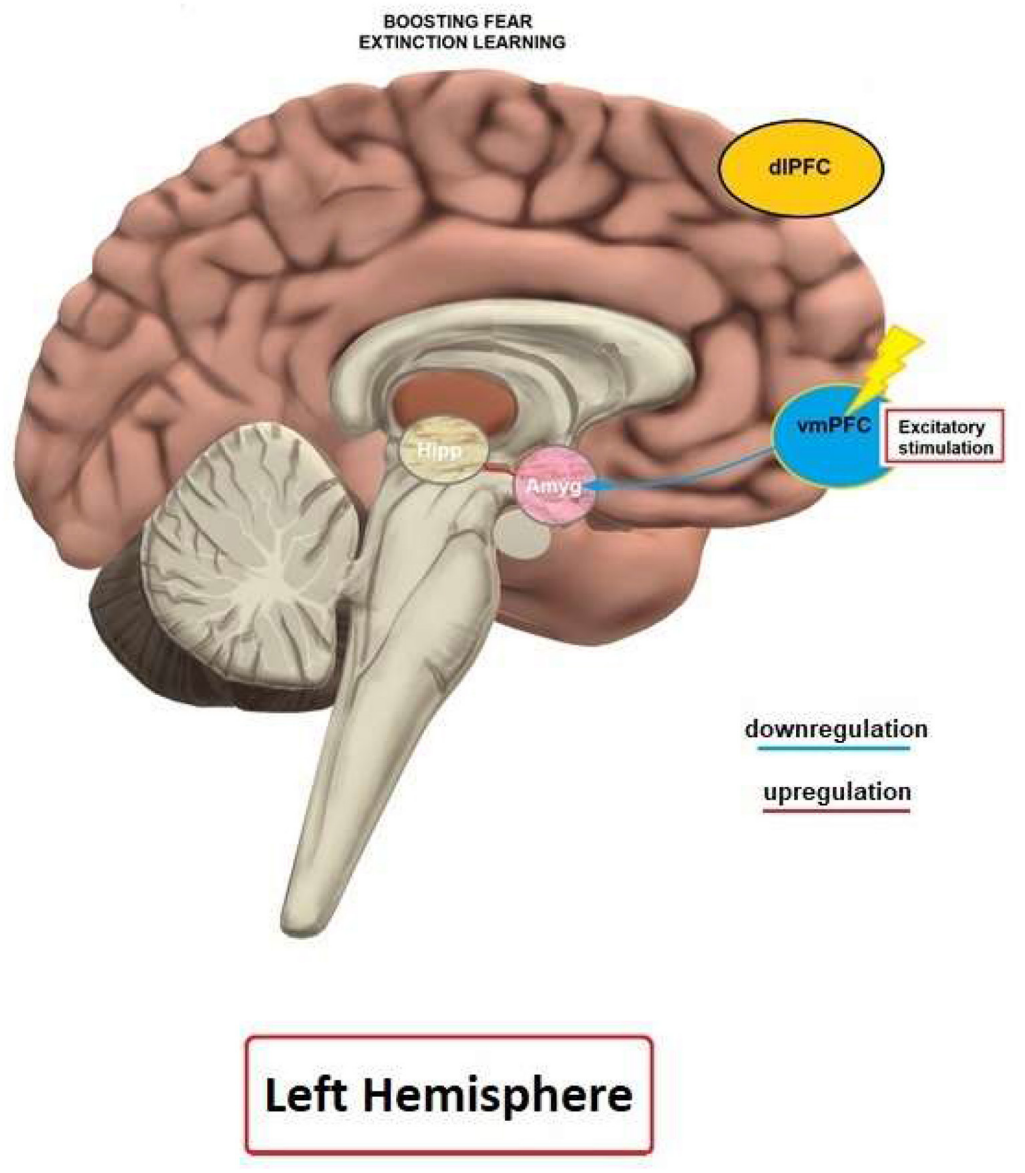
Model on how NIBS is thought to influence fear extinction memory. Excitatory NIBS over the left vmPFC downregulates the output of the central nucleus of the amygdala via activation of the intercalated nucleus, which in turn upregulates hippocampus, responsible for memory consolidation.

Regarding fear extinction, the results of the reviewed studies are in line with previous conclusions about the role of the vmPFC for top-down regulation of the amygdala (Milad et al., 2007), and that dysfunctions of vmPFC-amygdala connectivity may mediate the susceptibility to and/or maintenance of anxiety disorders (Milad et al., 2014). A model on how NIBS over the left vmPFC is suggested to improve fear extinction is shown in Figure 3. However, the field is only at its beginning, and some steps are required to make further advances.

**Figure 4:**
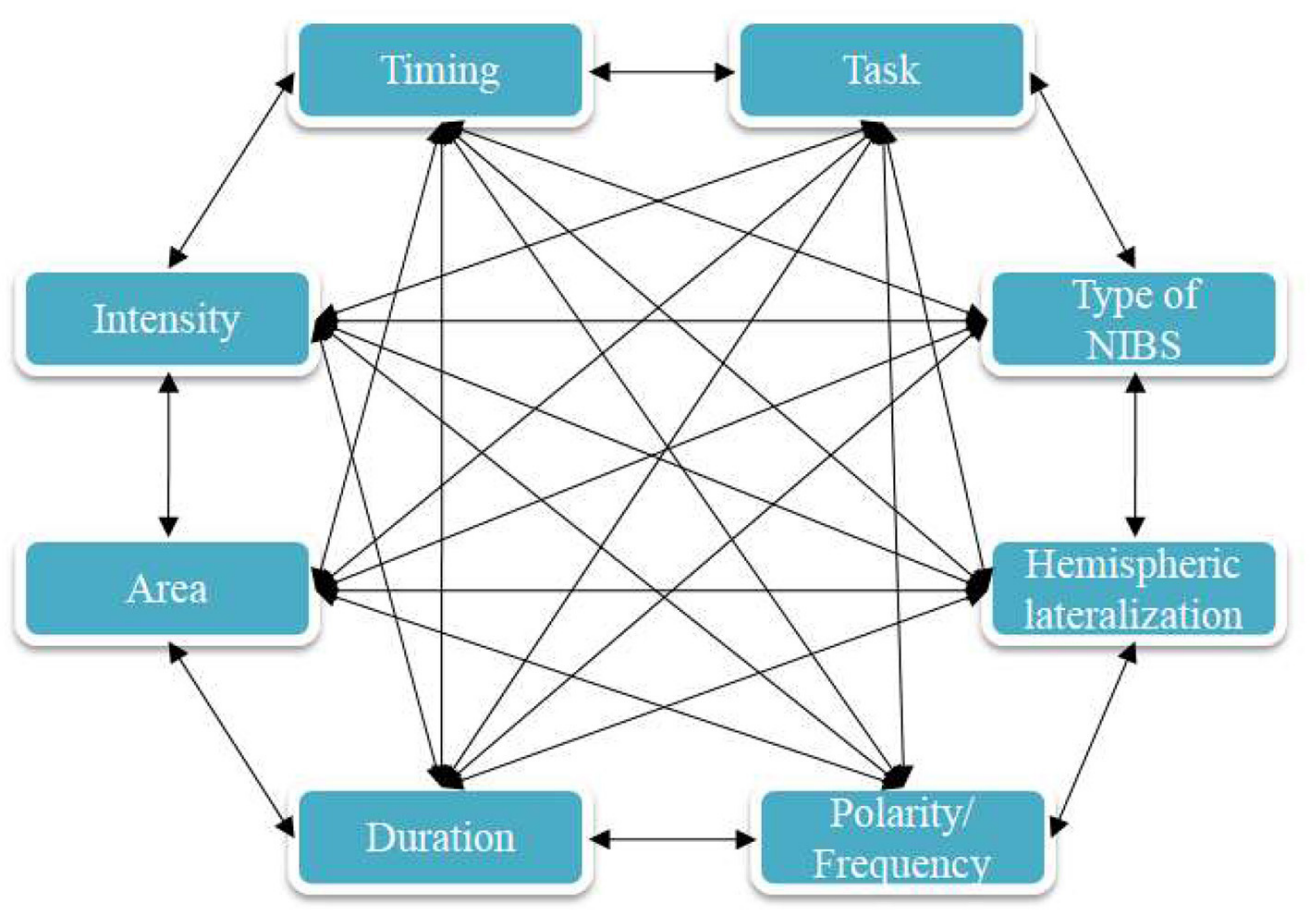
Complex and mutual relationships between various factors mediating the effects of NIBS methods on fear and extinction memory. *Type of NIBS*- refers to which NIBS method is applied, e.g., rTMS, tDCS; *Hemispheric lateralization* refers to whether stimulation is dominantly applied over the left or right hemisphere; *Polarity/frequency* refers to whether anodal or cathodal tDCS is applied, or low or high frequency rTMS; *Duration* refers to duration of the applied stimulation; *Area-* refers to which area is intended to be targeted for the stimulation; *Intensity* refers to the intensity of applied stimulation; *Timing* refers to whether stimulation is applied before, simultaneously or after the extinction/exposure; *Task-* refers to specifics of the fear conditioning/extinction protocol (e.g., reinforcement rate, modality of the US, using CS+ reminders).

Systematic studies are required to deliver information about the optimal area (including laterality), duration, intensity, polarity/frequency, and timing of stimulation. Here enhanced knowledge about physiological mechanisms of action of NIBS on fear and extinction memory would be helpful as a foundation for optimization; most of the studies in the field are purely behavioral. In this line, current NIBS protocols affect mostly superficial areas, and network effects on deeper structures remain largely unexplored. The adoption of other brain stimulation approaches such as transcranial focused ultrasound stimulation might help to overcome these limitations in future, in line with evidence from non-human primates (e.g., Folloni et al., 2019) suggesting that this method might be suited to modulate subcortical neural structures which are critical for fear processing and extinction. Another critical factor is the adoption of a more systematic procedure for fear conditioning/extinction protocols, as the variability of results in the examined literature might have been caused at least partially by task heterogeneities. Finally, the sample sizes in most of the studies in clinical populations are rather small, and future studies should employ larger groups. Large-scale studies are especially needed for protocols optimized for routine application in the clinical field. An overview of key variables to be systematically explored to investigate the effect of NIBS on fear memory/fear extinction learning is shown in Figure 4.

In summary, our review shows preliminary evidence that NIBS is a relevant method to modulate fear-related processes in humans. The main limitations of the available literature in the field, which should be addressed in future investigations, can be summarized as follows: (i) the low number of double blind, sham controlled studies (i.e., only about 14% of the available research); (ii) the absence of systematic titration of stimulation parameters such as duration, repetition, intensity and cortical target for optimization; (iii) the absence of systematic protocols which combine standard therapies with NIBS; (iv) a limited number of follow-up studies aiming at investigating the long-term effects of NIBS on fear memory and/or fear extinction learning processes; (v) the lack of mechanistic studies exploring the physiological foundation of NIBS effects.

## Data Availability Statement

The original contributions presented in the study are included in the article/supplementary material, further inquiries can be directed to the corresponding author/s.

## Author Contributions

CV and MN conceived the work. VM wrote the early version of the manuscript. FY, MS, and VM prepared tables. All authors revised and approved the final version of the manuscript.

## Conflict of Interest

MN is a member of the Scientific Advisory Boards of Neuroelectrics and NeuroDevice. The remaining authors declare that the research was conducted in the absence of any commercial or financial relationships that could be construed as a potential conflict of interest.
